# Use of the International IFOMPT Cervical Framework to inform clinical reasoning in postgraduate level physiotherapy students: a qualitative study using think aloud methodology

**DOI:** 10.1186/s12909-024-05399-x

**Published:** 2024-05-02

**Authors:** Katie L. Kowalski, Heather Gillis, Katherine Henning, Paul Parikh, Jackie Sadi, Alison Rushton

**Affiliations:** School of Physical Therapy, Western University, London, Ontario Canada

**Keywords:** International IFOMPT Cervical Framework, Clinical reasoning, Postgraduate students, Physiotherapy, Educational research, Qualitative research, Think aloud methodology

## Abstract

**Background:**

Vascular pathologies of the head and neck are rare but can present as musculoskeletal problems. The International Federation of Orthopedic Manipulative Physical Therapists (IFOMPT) Cervical Framework (Framework) aims to assist evidence-based clinical reasoning for safe assessment and management of the cervical spine considering potential for vascular pathology. Clinical reasoning is critical to physiotherapy, and developing high-level clinical reasoning is a priority for postgraduate (post-licensure) educational programs.

**Objective:**

To explore the influence of the Framework on clinical reasoning processes in postgraduate physiotherapy students.

**Methods:**

Qualitative case study design using think aloud methodology and interpretive description, informed by COnsolidated criteria for REporting Qualitative research. Participants were postgraduate musculoskeletal physiotherapy students who learned about the Framework through standardized delivery. Two cervical spine cases explored clinical reasoning processes. Coding and analysis of transcripts were guided by Elstein’s diagnostic reasoning components and the Postgraduate Musculoskeletal Physiotherapy Practice model. Data were analyzed using thematic analysis (inductive and deductive) for individuals and then across participants, enabling analysis of key steps in clinical reasoning processes and use of the Framework. Trustworthiness was enhanced with multiple strategies (e.g., second researcher challenged codes).

**Results:**

For all participants (*n* = 8), the Framework supported clinical reasoning using primarily hypothetico-deductive processes. It informed vascular hypothesis generation in the patient history and testing the vascular hypothesis through patient history questions and selection of physical examination tests, to inform clarity and support for diagnosis and management. Most participant’s clinical reasoning processes were characterized by high-level features (e.g., prioritization), however there was a continuum of proficiency. Clinical reasoning processes were informed by deep knowledge of the Framework integrated with a breadth of wider knowledge and supported by a range of personal characteristics (e.g., reflection).

**Conclusions:**

Findings support use of the Framework as an educational resource in postgraduate physiotherapy programs to inform clinical reasoning processes for safe and effective assessment and management of cervical spine presentations considering potential for vascular pathology. Individualized approaches may be required to support students, owing to a continuum of clinical reasoning proficiency. Future research is required to explore use of the Framework to inform clinical reasoning processes in learners at different levels.

**Supplementary Information:**

The online version contains supplementary material available at 10.1186/s12909-024-05399-x.

## Introduction

Musculoskeletal neck pain and headache are highly prevalent and among the most disabling conditions globally that require effective rehabilitation [1–4]. A range of rehabilitation professionals, including physiotherapists, assess and manage musculoskeletal neck pain and headache. Assessment of the cervical spine can be a complex process. Patients can present to physiotherapy with vascular pathology masquerading as musculoskeletal pain and dysfunction, as neck pain and/or headache as a common first symptom [5]. While vascular pathologies of the head and neck are rare [6], they are important considerations within a cervical spine assessment to facilitate the best possible patient outcomes [7]. The International IFOMPT (International Federation of Orthopedic Manipulative Physical Therapists) Cervical Framework (Framework) provides guidance in the assessment and management of the cervical spine region, considering the potential for vascular pathologies of the neck and head [8]. Two separate, but related, risks are considered: risk of misdiagnosis of an existing vascular pathology and risk of serious adverse event following musculoskeletal interventions [8]. 

The Framework is a consensus document iteratively developed through rigorous methods and the best contemporary evidence [8], and is also published as a Position Statement [7]. Central to the Framework are clinical reasoning and evidence-based practice, providing guidance in the assessment of the cervical spine region, considering the potential for vascular pathologies in advance of planned interventions [7, 8]. The Framework was developed and published to be a resource for practicing musculoskeletal clinicians and educators. It has been implemented widely within IFOMPT postgraduate (post-licensure) educational programs, influencing curricula by enabling a comprehensive and systemic approach when considering the potential for vascular pathology [9]. Frequently reported curricula changes include an emphasis on the patient history and incorporating Framework recommended physical examination tests to evaluate a vascular hypothesis [9]. The Framework aims to assist musculoskeletal clinicians in their clinical reasoning processes, however no study has investigated students’ use of the Framework to inform their clinical reasoning.

Clinical reasoning is a critical component to physiotherapy practice as it is fundamental to assessment and diagnosis, enabling physiotherapists to provide safe and effective patient-centered care [10]. This is particularly important for postgraduate physiotherapy educational programs, where developing a high level of clinical reasoning is a priority for educational curricula [11] and critical for achieving advanced practice physiotherapy competency [12–15]. At this level of physiotherapy, diagnostic reasoning is emphasized as an important component of a high level of clinical reasoning, informed by advanced use of domain-specific knowledge (e.g., propositional, experiential) and supported by a range of personal characteristics (e.g., adaptability, reflective) [12]. Facilitating the development of clinical reasoning improves physiotherapist’s performance and patient outcomes [16], underscoring the importance of clinical reasoning to physiotherapy practice. Understanding students’ use of the Framework to inform their clinical reasoning can support optimal implementation of the Framework within educational programs to facilitate safe and effective assessment and management of the cervical spine for patients.

### Objective

To explore the influence of the Framework on the clinical reasoning processes in postgraduate level physiotherapy students.

## Methods

### Design

Using a qualitative case study design, think aloud case analyses enabled exploration of clinical reasoning processes in postgraduate physiotherapy students. Case study design allows evaluation of experiences in practice, providing knowledge and accounts of practical actions in a specific context [17]. Case studies offer opportunity to generate situationally dependent understandings of accounts of clinical practice, highlighting the action and interaction that underscore the complexity of clinical decision-making in practice [17]. This study was informed by an interpretive description methodological approach with thematic analysis [18, 19]. Interpretive description is coherent with mixed methods research and pragmatic orientations [20, 21], and enables generation of evidence-based disciplinary knowledge and clinical understanding to inform practice [18, 19, 22]. Interpretive description has evolved for use in educational research to generate knowledge of educational experiences and the complexities of health care education to support achievement of educational objectives and professional practice standards [23]. The COnsolidated criteria for REporting Qualitative research (COREQ) informed the design and reporting of this study [24]. 

### Research team

All research team members hold physiotherapy qualifications, and most hold advanced qualifications specializing in musculoskeletal physiotherapy. The research team is based in Canada and has varying levels of academic credentials (ranging from Clinical Masters to PhD or equivalent) and occupations (ranging from PhD student to Director of Physical Therapy). The final author (AR) is also an author of the Framework, which represents international and multiprofessional consensus. Authors HG and JS are lecturers on one of the postgraduate programs which students were recruited from. The primary researcher and first author (KK) is a US-trained Physical Therapist and Postdoctoral Research Associate investigating spinal pain and clinical reasoning in the School of Physical Therapy at Western University. Authors KK, KH and PP had no prior relationship with the postgraduate educational programs, students, or the Framework.

### Study setting

Western University in London, Ontario, Canada offers a one-year Advanced Health Care Practice (AHCP) postgraduate IFOMPT-approved Comprehensive Musculoskeletal Physiotherapy program (CMP) and a postgraduate Sport and Exercise Medicine (SEM) program. Think aloud case analyses interviews were conducted using Zoom, a viable option for qualitative data collection and audio-video recording of interviews that enables participation for students who live in geographically dispersed areas across Canada [25]. Interviews with individual participants were conducted by one researcher (KK or KH) in a calm and quiet environment to minimize disruption to the process of thinking aloud [26]. 

### Participants

AHCP postgraduate musculoskeletal physiotherapy students ≥ 18 years of age in the CMP and SEM programs were recruited via email and an introduction to the research study during class by KK, using purposive sampling to ensure theoretical representation. The purposive sample ensured key characteristics of participants were included, specifically gender, ethnicity, and physiotherapy experience (years, type). AHCP students must have attended standardized teaching about the Framework to be eligible to participate. Exclusion criteria included inability to communicate fluently in English. As think-aloud methodology seeks rich, in-depth data from a small sample [27], this study sought to recruit 8–10 AHCP students. This range was informed by prior think aloud literature and anticipated to balance diversity of participant characteristics, similarities in musculoskeletal physiotherapy domain knowledge and rich data supporting individual clinical reasoning processes [27, 28]. 

### Learning about the IFOMPT Cervical Framework

CMP and SEM programs included standardized teaching of the Framework to inform AHCP students’ clinical reasoning in practice. Delivery included a presentation explaining the Framework, access to the full Framework document [8], and discussion of its role to inform practice, including a case analysis of a cervical spine clinical presentation, by research team members AR and JS. The full Framework document that is publicly available through IFOMPT [8] was provided to AHCP students as the Framework Position Statement [7] was not yet published. Discussion and case analysis was led by AHCP program leads in November 2021 (CMP, including research team member JS) and January 2022 (SEM).

### Think aloud case analyses data collection

Using think aloud methodology, the analytical processes of how participants use the Framework to inform clinical reasoning were explored in an interview with one research team member not involved in AHCP educational programs (KK or KH). The think aloud method enables description and explanation of complex information paralleling the clinical reasoning process and has been used previously in musculoskeletal physiotherapy [29, 30]. It facilitates the generation of rich verbal [27]as participants verbalize their clinical reasoning protocols [27, 31]. Participants were aware of the aim of the research study and the research team’s clinical and research backgrounds, supporting an open environment for depth of data collection [32]. There was no prior relationship between participants and research team members conducting interviews.

Participants were instructed to think aloud their analysis of two clinical cases, presented in random order (Supplementary 1). Case information was provided in stages to reflect the chronology of assessment of patients in practice (patient history, planning the physical examination, physical examination, treatment). Use of the Framework to inform clinical reasoning was discussed at each stage. The cases enabled participants to identify and discuss features of possible vascular pathology, treatment indications and contraindications/precautions, etc. Two research study team members (HG, PP) developed cases designed to facilitate and elicit clinical reasoning processes in neck and head pain presentations. Cases were tested against the research team to ensure face validity. Cases and think aloud prompts were piloted prior to use with three physiotherapists at varying levels of practice to ensure they were fit for purpose.

Data collection took place from March 30-August 15, 2022, during the final terms of the AHCP programs and an average of 5 months after standardized teaching about the Framework. During case analysis interviews, participants were instructed to constantly think aloud, and if a pause in verbalizations was sustained, they were reminded to “keep thinking aloud” [27]. As needed, prompts were given to elicit verbalization of participants’ reasoning processes, including use of the Framework to inform their clinical reasoning at each stage of case analysis (Supplementary 2). Aside from this, all interactions between participants and researchers minimized to not interfere with the participant’s thought processes [27, 31]. When analysis of the first case was complete, the researcher provided the second case, each lasting 35–45 min. A break between cases was offered. During and after interviews, field notes were recorded about initial impressions of the data collection session and potential patterns appearing to emerge [33]. 

### Data analysis

**Fig. 1:**
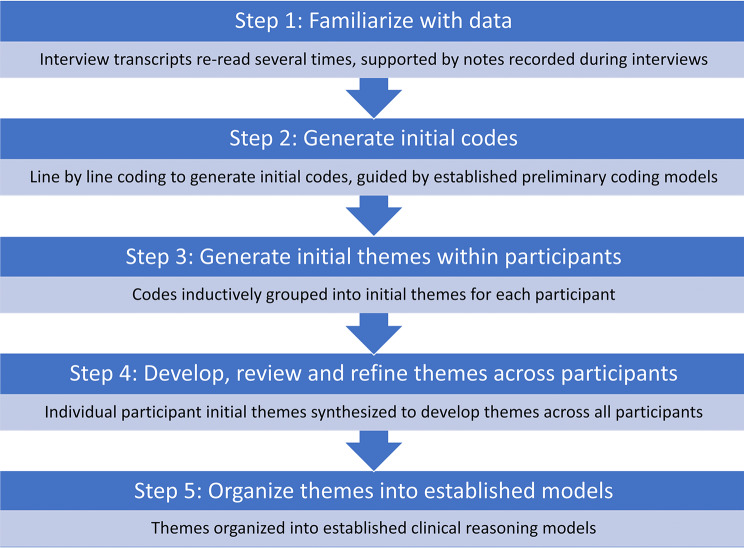
Data analysis steps

Data from think aloud interviews were analyzed using thematic analysis [30, 34], facilitating identification and analysis of patterns in data and key steps in the clinical reasoning process, including use of the Framework to enable its characterization (Fig. 1). As established models of clinical reasoning exist, a hybrid approach to thematic analysis was employed, incorporating inductive and deductive processes [35], which proceeded according to 5 iterative steps: [34]

1. Familiarize with data: Audio-visual recordings were transcribed verbatim by a physiotherapist external to the research team. All transcripts were read and re-read several times by one researcher (KK), checking for accuracy by reviewing recordings as required. Field notes supported depth of familiarization with data.
2. Generate initial codes: Line-by-line coding of transcripts by one researcher (KK) supported generation of initial codes that represented components, patterns and meaning in clinical reasoning processes and use of the Framework. Established preliminary coding models were used as a guide. Elstein’s diagnostic reasoning model [36] guided generating initial codes of key steps in clinical reasoning processes (Table 1a) [29, 36]. Leveraging richness of data, further codes were generated guided by the Postgraduate Musculoskeletal Physiotherapy Practice model, which describes masters level clinical practice (Table 1b) [12]. Codes were refined as data analysis proceeded. All codes were collated within participants along with supporting data.
3. Generate initial themes within participants: Coded data was inductively grouped into initial themes within each participant, reflecting individual clinical reasoning processes and use of the Framework. This inductive stage enabled a systematic, flexible approach to describe each participant’s unique thinking path, offering insight into the complexities of their clinical reasoning processes. It also provided a comprehensive understanding of the Framework informing clinical reasoning and a rich characterization of its components, aiding the development of robust, nuanced insights [35, 37, 38]. Initial themes were repeatedly revised to ensure they were grounded in and reflected raw data.
4. Develop, review and refine themes across participants: Initial themes were synthesized across participants to develop themes that represented all participants. Themes were reviewed and refined, returning to initial themes and codes at the individual participant level as needed.
5. Organize themes into established models: Themes were deductively organized into established clinical reasoning models; first into Elstein’s diagnostic reasoning model, second into the Postgraduate Musculoskeletal Physiotherapy Practice model to characterize themes within each diagnostic reasoning component [12, 36].

**Table 1:** Definitions of initial codes and examples

Codes	Definition	Example quote
**1a. Codes using Elstein’s diagnostic reasoning model components** ^a^		
Cue acquisition	Gathering information (cues) not linked to a specific hypothesis	*“If it’s a sharp pain or a headache, that is one that’s never been experienced before?”* Student 4
Hypothesis generation	Using and / or interpreting cues to generate diagnostic hypotheses	*“General hypotheses right now would be whiplash, concussion”* Student 2
Cue evaluation	Interpreting cues and assessing the value in relation to hypotheses	*“Her blood pressure seems normal, but her resting heart rate seems high for someone who’s been so active.”* Student 3
Hypothesis evaluation	Forming a judgement as to the value of hypotheses and decision as to the most plausible hypothesis	*“All supporting the mechanical convergent neck pain and C4/5”* Student 1
Treatment	All aspects of treatment, including referral for further investigations, and planning / implementing treatment with physiotherapy interventions	*“I would slowly engage her to be more active”* Student 6
**1b. Codes using Postgraduate Musculoskeletal Physiotherapy Practice model components** [12]		
Clinical reasoning	Cognitive processes used to analyze and interpret case information, and formulate a diagnosis or treatment plan	*“Clear pattern of convergent mechanical neck pain.doesn’t seem to be any neuropathic contributors.”* Student 1
Knowledge	All types of knowledge (e.g., propositional, experiential)	*“When you look at the IFOMPT Framework on the risk factors, you know, smoking, increased blood pressure, and increased cholesterol levels and these are the risk factors for non-dissecting type of vascular events”* Student 5
Personal characteristics	Personal characteristics of students (e.g., adaptability, reflection)	Reflection: *“They say like palpation and auscultation. But honestly, I don’t really do that in clinic…I wouldn’t say like, I am good at determining if there’s a difference.”* Student 7

^a^ Elstein’s diagnostic reasoning model components (cue acquisition, hypothesis generation, cue evaluation, hypothesis evaluation) and treatment, an adaptation previously used in physiotherapy research [29, 36].

### Trustworthiness of findings

The research study was conducted according to an *a priori* protocol and additional steps were taken to establish trustworthiness of findings [39]. Field notes supported deep familiarization with data and served as a means of data source triangulation during analysis [40]. One researcher coded transcripts and a second researcher challenged codes, with codes and themes rigorously and iteratively reviewed and refined. Frequent debriefing sessions with the research team, reflexive discussions with other researchers and peer scrutiny of initial findings enabled wider perspectives and experiences to shape analysis and interpretation of findings. Several strategies were implemented to minimize the influence of prior relationships between participants and researchers, including author KK recruiting participants, KK and KH collecting/analyzing data, and AR, JS, HG and PP providing input on de-identified data at the stage of synthesis and interpretation.

## Results

### Participants

Nine AHCP postgraduate level students were recruited and participated in data collection. One participant was withdrawn because of unfamiliarity with the standardized teaching session about use of the Framework (no recall of session), despite confirmation of attendance. Data from eight participants were used for analysis (CMP: *n* = 6; SEM: *n* = 2; Table 2), which achieved sample size requirements for think aloud methodology of rich and in-depth data [27, 28]. 

**Table 2:** Characteristics of AHCP student participants

Gender, n (Women: Men)	4: 4
Age, years (Median, Range)	33.5, 28–43
Ethnicity (n)	Caucasian: 3 Chinese: 1 Eastern European: 1 Hispanic: 1 Mixed (Chinese, Caucasian): 1 South Asian: 1
County of entry to practice training (n)	Canada: 6 India: 1 Netherlands: 1
Clinical practice experience, years (Median, Range)	7.5, 3–14
Completed postgraduate education (n)	Thesis-based MSc: 2 FCAMPT: 1 Continuing professional development courses: Advanced orthopedics: 2 Acupuncture: 3 Concussion: 2 Dry needling: 1 Intramuscular stimulation: 1 Mulligan technique: 1 Soft tissue release: 1 Spinal manipulation: 1 Sports performance training: 1
Clinical practice setting (n)	Outpatient private practice: 8
Clinical population (n)	Adult musculoskeletal populations: 7 Adolescent / young adult sports: 1

FCAMPT, Fellow of the Canadian Academy of Manipulative Physiotherapy; MSc, Master of Science

### Diagnostic reasoning components

Informed by the Framework, all components of Elstein’s diagnostic reasoning processes [36] were used by participants, including use of treatment with physiotherapy interventions to aid diagnostic reasoning. An illustrative example is presented in Supplement 3. Clinical reasoning used primarily hypothetico-deductive processes reflecting a continuum of proficiency, was informed by deep Framework knowledge and breadth of prior knowledge (e.g., experiential), and supported by a range of personal characteristics (e.g., justification for decisions).

#### Cue acquisition

All participants sought to acquire additional cues early in the patient history, and for some this persisted into the medical history and physical examination. Cue acquisition enabled depth and breadth of understanding patient history information to generate hypotheses and factors contributing to the patient’s pain experience (Table 3). All participants asked further questions to understand details of the patients’ pain and their presentation, while some also explored the impact of pain on patient functioning and treatments received to date. There was a high degree of specificity to questions for most participants. Ongoing clinical reasoning processes through a thorough and complete assessment, even if the patient had previously received treatment for similar symptoms, was important for some participants. Cue acquisition was supported by personal characteristics including a patient-centered approach (e.g., understanding the patient’s beliefs about pain) and one participant reflected on their approach to acquiring patient history cues.

**Table 3:** Cue acquisition themes and illustrative quotes

Construct	Cue acquisition themes and illustrative quotes
Clinical reasoning	All participants asked further questions early in the patient history to understand details of the patient’s pain and their presentation, with a high degree of specificity for most participants: “*I’d want to know the intensity of the pain, the time of the pain. So like, when they’re resting at night, when they’re waking up in the morning, when they’re standing. So like all those types of like, the qualities of the pain? Is it sharp? Does it radiate anywhere else? Is it, is there seem to be a mechanical, or like a movement related increase in pain? Or does it resolve with rest?”* Student 4 The impact of pain on patient functioning was explored by some participants: “*Knowing…if sleep has been affected too, because of this. And just overall, how she feels her function is affected?…Is she pushing through her work and just continuing to do everything she has been doing? Or is she having to really modify at this point?…And more socially to how this is affecting her life outside of work too. Is there certain things that she’s having to give up?”* Student 2 Treatments received to date were queried by some participants: “*And if she’s checked with another health practitioner before, if she had any chiro, massage treatment before they saw me because it’s been three or four months now.”* Student 6 Ongoing reasoning through a thorough and complete clinical assessment was important for some participants, even if the patient had previously received treatment with resolution of symptoms: * “When patients, you know, had success one time at physio for neck pain, sometimes they just come here first expecting the same thing. So, we want to just make sure that we’re doing still a thorough assessment and not assuming it’s the same thing and just treating the same way that we previously did.”* Student 7
Personal characteristics	Understanding a broad range of potential contributing factors to the patient’s experience of pain was important for most participants, highlighting a patient centered approach: * “I’m wondering what might have happened three to four months ago, that changed in her life that brought on this neck pain. I’m really curious if she changed jobs, or maybe change setups or if there was like a stressful situation in her life that maybe brought this on.”* Student 3 Cue acquisition persisted into the patient history and physical examination for some participants to understand the patient’s beliefs about pain and success of previous treatments received: * “I would ask her what she believes is causing the symptoms at this point…And then asking her maybe you know, if she was given exercises in the past did she did she try to do them and…did it alter what she was feeling in either positive or negative way?”* Student 2 One student reflected on their approach to acquiring cues in a patient history: * “I think a lot of the times I sit, and I like to listen, I don’t want to lead…looking for them to say something that that triggers something in me to go further down somewhere…I don’t want to frighten this patient as well. I think so I would be more listening at this point…I don’t want to bias and lead with questions.”* Student 4

#### Hypothesis generation

Participants generated an average of 4.5 hypotheses per case (range: 2–8) and most hypotheses (77%) were generated rapidly early in the patient history. Knowledge from the Framework about patient history features of vascular pathology informed vascular hypothesis generation in the patient history for all participants in both cases (Table 4). Vascular hypotheses were also generated during the past medical history, where risk factors for vascular pathology were identified and interpreted by some participants who had high levels of suspicion for cervical articular involvement. Non-vascular hypotheses were generated during the physical examination by some participants to explain individual physical examination or patient history cues. Deep knowledge of the patient history section in the Framework supported high level of cue identification and interpretation for generating vascular hypotheses. Initial hypotheses were prioritized by some participants, however the level of specificity of hypotheses varied.

**Table 4:** Hypothesis generation themes and illustrative quotes

Construct	Hypothesis generation themes and illustrative quotes
Clinical reasoning	The Framework patient history section (risk factors, symptoms, signs of vascular pathology) supported a high level of cue identification and interpretation for generating a vascular hypothesis for all but one participant: “S*he’s younger which is always good, and she seems pretty active, but definitely that trauma…it does make me already have a little bit of a red flag…It’s also a good thing that she could resume skiing…Starting to get a right side of neck pain and tightness, which could just be a whiplash injury or neck strain. But given her trauma…and also the headache, so it is only one-sided headaches. So it makes me wonder okay, could be more of like a referral type of headache or a…tension type of headache or a whiplash type of headache as opposed to something vascular, like a migraine type of thing.”* Student 3 Vascular hypotheses were also generated during the past medical history, where risk factors for vascular pathology were identified and interpreted by some participants who had high levels of suspicion for cervical articular involvement: * “There’s some little concerns about some of the medical history, hypertension, diabetes, obese, depression, like high cholesterol…smoking through young adulthood…so it’s possible that the neck pain is coming from the arteries in the neck area…it might be a secondary hypothesis…I don’t think it changes my primary* [facet joints].*”* Student 6 Non-vascular hypotheses were also generated during the physical examination by some participants to explain individual physical examination or patient history cues: * “P3 is kind of an awkward location here with it being she’s thinking it’s sinus, but we didn’t really talk about TMJ or jaw…is it something that’s referring across?”* Student 2 Initial hypotheses generated were prioritized by some participants: * “Fracture is not my first primary one. But it’s there. The second one.it’s arterial problem, dissection.”* Student 6 The level of specificity in initial hypotheses generated varied: * “Mechanical pain at motion segments C4/5…with somatic referral”* Student 5 * “I’m thinking more muscular, postural related stuff.”* Student 8
Knowledge	Knowledge from the Framework about patient history features (risk factors, symptoms, signs) of vascular pathology informed generating a vascular hypothesis for all participants: * “The numbers from the Framework again, that’s how I find I like to use it when I’m assessing risk and this one, so we know she’s had like a recent history of trauma. She’s starting to develop like signs of like unsteadiness…So, we’re starting to get into some of those higher risk factors for that like dissecting stroke which is listed in like the higher prevalence risk factors from the Framework.”* Student 1 Deep Framework knowledge was integrated with a breadth of prior knowledge (e.g., experiential): * “This one’s a lot more of like, practice experience with like, okay, it’s very much that like setting those pain drivers. So it’s like it’s very localized. It’s very movement dependent, clear, aggravating…the only thing that Framework, I think, really in this case is all of those risk factors that are on those lists for dissection are not here, other than maybe the light-headedness but even that that wasn’t high up on those risk factor lists anyways.” Student 1*

#### Cue evaluation

All participants evaluated cues throughout the patient history and physical examination in relationship to hypotheses generated, indicating use of hypothetico-deductive reasoning processes (Table 5). Framework knowledge of patient history features of vascular pathology was used to test vascular hypotheses and aid differential diagnosis. The patient history section supported high level of cue identification and interpretation of patient history features for all but one participant, and generation of further patient history questions for all participants. The level of specificity of these questions was high for all but one participant. Framework knowledge of recommended physical examination tests, including removal of positional testing, supported planning a focused and prioritized physical examination to further test vascular hypotheses for all participants. No participant indicated intention to use positional testing as part of their physical examination. Treatment with physiotherapy interventions served as a form of cue evaluation, and cues were evaluated to inform prognosis for some participants. At times during the physical examination, some participants demonstrated occasional errors or difficulty with cue evaluation by omitting key physical exam tests (e.g., no cranial nerve assessment despite concerns for trigeminal nerve involvement), selecting physical exam tests in advance of hypothesis generation (e.g., cervical spine instability testing), difficulty interpreting cues, or late selection of a physical examination test. Cue acquisition was supported by a range of personal characteristics. Most participants justified selection of physical examination tests, and some self-reflected on their ability to collect useful physical examination information to inform selection of tests. Precaution to the physical examination was identified by all participants but one, which contributed to an adaptable approach, prioritizing patient safety and comfort. Critical analysis of physical examination information aided interpretation within the context of the patient for most participants.

**Table 5:** Cue evaluation themes and illustrative quotes

Construct	Cue evaluation themes and illustrative quotes
Clinical reasoning	The Framework patient history section (risk factors, symptoms, signs of vascular pathology) supported a high level of cue identification and interpretation to test a vascular hypothesis for all but one participant: * “There are definitely a lot more things that are jumping out at me…And it would be informed by the IFOMPT Framework. So for example, this pain in through P3, which is in the distribution of the cranial nerve five, trigeminal nerve…it’s a constant severe pain. And also she’s feeling unsteady on the stairs, also is a red flag for kind of like an ataxia type of thing. And that’s also informed by this IFOMPT Framework. I’m thinking about the tables….the tables, for me, were really helpful to kind of visualize, okay, dissection, injury, trauma is like a huge one.”* Student 3 Further patient history questions were asked by all participants to test their vascular hypothesis and aid differential diagnosis. The level of specificity of these questions was high for all but one participant: * “I would ask more related to the symptoms of the cranial nerves. I would ask if she’s having difficulty swallowing, any nausea vomiting sensation…difficulty talking….any blurry vision, double vision, and does she have any spinning episodes or dizziness, or unsteadiness she has mentioned. Does she have any other issues with the fullness in the ear or ringing in the ear?”* Student 5 * “Ask I guess if they’re noticing any weakness, any vision changes. I guess slurred speech or anything like that…Those are kind of I guess cranial nerve questions.”* Student 8 Framework recommended physical examination tests supported planning a focused and prioritized physical examination to test the vascular hypothesis for all participants: * “I would start like that, inspection, pulses, blood pressure…and then cranial nerves. If everything is normal and clean, I would continue with my assessment with range of motion, strength test, and go from there.”* Student 6 No participant used positional testing to test the vascular hypothesis: * “We do know, with the Framework, that you know, the positional testing isn’t really what we want to be basing our reasoning off.”* Student 7 Treatment with physiotherapy interventions also served as cue evaluation during the physical examination for some participants: “*Maybe even try to get her on a bike or a treadmill. That’ll be more kind of assessment and treatment together really.”* Student 1 Cues were also evaluated to inform prognosis for some participants: * “That also kind of informs my prognosis of what might be, of how long it might take for her to get her neck pain better, even if it is just a mechanical issue.”* Student 3 At times during the physical examination, some participants demonstrated occasional errors and difficulty, for example difficulty interpreting cues and late identification of a physical examination test to perform: * “Because of P3 and how I’m not 100% sure what might be causing that.”* Student 3 * “I forgot throughout this whole thing that you could also auscultate…the carotid arteries.”* Student 8
Knowledge	Knowledge from the Framework about patient history features (risk factors, symptoms, signs of vascular pathology) supported testing a vascular hypothesis during the patient history for all participants: * “The Framework just has some really nicely laid out tables where…it does go through risk factors. So associated with just investigating, again, dissecting versus non dissecting events. And so just looking at the hypertension there, and the high cholesterol, and I mean, she did smoke occasionally, she’s not an active smoker anymore. But that is also something that is in within the Framework is a pretty significant risk factor. So that’s where it’s helping me well, just guiding, having those tables laid out nicely.”* Student 2 Framework knowledge of recommended physical examination tests supported planning a focused and prioritized physical examination to test the vascular hypothesis for all participants: * “The Framework is, is recommending, okay, if you’re suspecting let’s do the blood pressure first see, is it is it high?… And then recommending…if these blood pressure readings are abnormally high for the client, okay, let’s do a cranial nerve assessment and see is that is that showing anything abnormal?”* Student 2 This also included knowledge from the Framework about the removal of positional testing, which all participants did not select to test the vascular hypothesis: * “The Framework says, that doesn’t add value, and is not an indicator of vascular event.”* Student 5
Personal characteristics	Justification for selection of physical examination tests occurred in most participants: * “I didn’t do any sensation tests because I don’t expect any nerve involvement right now. Reflexes either. I just did a myotome test. It’s also to give me some overall idea about her strength in her upper extremities.”* Student 6 Self-reflection on ability to collect useful physical examination information informed selection of physical examination tests for some participants: * “I would want deep tendon or lower body information for me because I think I’m a little bit better at gathering that information personally.”* Student 4 Critical analysis of physical examination cues aided interpretation within the context of the patient for most participants: * “If they were someone that was normally 90 over 60, which a lot of active people would hang around there, this might be high for them. But that’s in general, are very normal within the normal range of, of blood pressure.”* Student 4 Precaution to the physical examination was identified by all participants but one, which contributed to an adaptable approach to the physical examination, prioritizing patient safety and comfort: * “I would keep it in patient’s tolerance level, she’s been dealing with this pain for six days now. She knows what’s tolerable and what’s not. So I would go make that guide my examination for now.”* Student 6

#### Hypothesis evaluation

All participants used the Framework to evaluate their hypotheses throughout the patient history and physical examination, continuously shifting their level of support for hypotheses (Table 6, Supplement 4). This informed clarity in the overall level of suspicion for vascular pathology or musculoskeletal diagnoses, which were specific for most participants. Response to treatment with physiotherapy interventions served as a form of hypothesis evaluation for most participants who had low level suspicion for vascular pathology, highlighting ongoing reasoning processes. Hypotheses evaluated were prioritized by ranking according to level of suspicion by some participants. Difficulties weighing patient history and physical examination cues to inform judgement on overall level of suspicion for vascular pathology was demonstrated by some participants who reported that incomplete physical examination data and not being able to see the patient contributed to difficulties. Hypothesis evaluation was supported by the personal characteristic of reflection, where some students reflected on the Framework’s emphasis on the patient history to evaluate a vascular hypothesis.

**Table 6:** Hypothesis evaluation themes and illustrative quotes

Construct	Hypothesis evaluation themes and illustrative quotes
Clinical reasoning	Hypothesis evaluation throughout the patient history and physical examination informed clarity in the overall level of suspicion for vascular pathology or musculoskeletal diagnoses, which were specific diagnoses for most participants: * “The range of motion…it’s giving a very clear restriction in her left facet joints…the first hypothesis we talked about now is confirming closer…I can now simply call it a convergent pattern. And, and I was calling it more of a motion segment dysfunction. I’m going to even go further and say gives me more evidence to go more into the joint itself.”* Student 5 * “At this point, yeah, more investigation would need to be done…the risk is too high to just begin with a, your typical physiotherapy management.”* Student 2 Response to treatment with physiotherapy interventions also served as a form of hypothesis evaluation for most participants who had low level suspicion for vascular pathology, highlighting ongoing reasoning processes: * “I find that’s where I get a lot of the information is on that second visit, like if we were just started with that, and then she comes back and like oh, it is starting to feel a little bit nicer, then I’m really starting to think okay, I don’t think there’s something vascular going on.”* Student 1 Hypotheses evaluated were ranked by some participants according to level of suspicion: * “Cervicogenic headache…that would be kind of primary. The other differential would be…generalized whiplash and…my third would be…based on the trauma…the vascular is still there.”* Student 7 Some participants demonstrated difficulties weighing patient history and physical examination cues to inform judgement on overall level of suspicion for vascular pathology: * “It’s a little bit of a grey area right now…she has a higher risk of having this dissection stroke potentially and given her like location of pain. Yeah, it does make me question. If yeah, I just don’t want to miss anything by weighing these risk factors incorrectly. So I’d rather just be safer and just say okay, let’s clear this first.”* Student 3 Contributing factors to difficulties with hypothesis evaluation were incomplete physical examination data and not being able to see the patient, which contributes to a gut feeling: * “I would still be interested in, see how the nerve roots are okay, but what are the cranial nerves?…And also just the unsteadiness too and wanting to investigate that a little bit further.”* Student 2 * “I feel that there has been insufficient again that I would like to know if that balance, was it ataxic thing? I don’t, I can’t see the person…the gut feeling does, when someone’s in front of you, contribute to my overall process.”* Student 4
Personal characteristics	Some students reflected on the Framework’s emphasis on the patient history to evaluate a vascular hypothesis: * “I’m just very much more inclined to consider cardiovascular risk factors, and then how they relate to symptom reporting versus what we were taught initially, which was that if you put the neck in a certain position, you could tear somebody’s vertebral artery.”* Student 4

#### Treatment

The Framework supported all participants in clinical reasoning related to treatment (Table 7). Treatment decisions were always linked to the participant’s overall level of suspicion for vascular pathology or musculoskeletal diagnosis. Framework knowledge supported participants with high level of suspicion for vascular pathology to refer for further investigations. Participants with a musculoskeletal diagnosis kept the patient for physiotherapy interventions. The Framework patient history section supported patient education about symptoms of vascular pathology and safety netting for some participants. Framework knowledge influenced informed consent processes and risk-benefit analysis to support the selection of musculoskeletal physiotherapy interventions, which were specific and prioritized for some participants. Less Framework knowledge related to treatment was demonstrated by some students, generating unclear recommendations regarding the urgency of referral and use of the Framework to inform musculoskeletal physiotherapy interventions. Treatment was supported by a range of personal characteristics. An adaptable approach that prioritized patient safety and was supported by justification was demonstrated in all participants except one. Shared decision-making enabled the selection of physiotherapy interventions, which were patient-centered (individualized, considered whole person, identified future risk for vascular pathology). Communication with the patient’s family doctor facilitated collaborative patient-centered care for most participants.

**Table 7:** Treatment themes and illustrative quotes

Construct	Treatment themes and illustrative quotes
Clinical reasoning	Participants with a high level of suspicion for vascular pathology referred the patient for further investigations to the emergency room, or in one instance to the patient’s family doctor. This was justified by the doctor’s historical knowledge and the patient’s current status: * “I’m leaning towards sending her to the family doctor waiting for two days. Since he knows her history. She’s still functioning, her vital seem okay.”* Student 3 Participants with a musculoskeletal diagnosis kept the patient for physiotherapy interventions. The Framework patient history section supported patient education of symptoms of vascular pathology and safety netting for some participants: * “I would still safety net her, you know, based on your trauma, we just want you to be monitoring for any symptoms. And if you have those, so more of like the vascular symptoms, we want you to report to emerg* [emergency department] *and not wait, you know, two days to see your family doctor.”* Student 7 Physiotherapy interventions were specific for some participants: * “I’m already thinking I’ll be directing some manual therapy at the C* [Cervical] *four, five segment, because that’s kind of where I’m thinking the root of the problem is.”* Student 1 Prioritization of physiotherapy interventions was important for some participants: * “Simple range of motion exercises…that would be my primary to help with her mobility and pain management.”* Student 5
Knowledge	Framework knowledge supported the decision about the need for referral for all participants who had a high level of suspicion of vascular pathology: * “It says like, okay, if, if their subjective is, you know, leading you towards that, then you don’t want to do anything, then refer. And then if their assessment is positive, refer. If it’s not, then do some of your more, you know, manual therapy testing or movement, active range. If that’s bad, then refer, but if not, then kind of continue with treatment, and just keep a close eye and evaluate.”* Student 8 In advanced of planned musculoskeletal physiotherapy interventions, Framework knowledge influenced informed consent considering the potential for vascular pathology for some participants: * “The framing of, of informed consent is maybe a little bit different for this person…That would probably come from the Framework.”* Student 4 Framework knowledge of risks and benefits of musculoskeletal physiotherapy interventions supported selection of physiotherapy interventions for most participants: * “The Framework, like say, it’s that, like cervical manipulation is still pretty low risk.”* Student 1 Some students demonstrated a lower level of Framework knowledge related to treatment, generating unclear recommendations regarding urgency of referral and use of the Framework to inform planning musculoskeletal physiotherapy interventions: * “I know I’m not really that familiar with the Framework to really know what it says about 911, or like emergency room, or is it okay to wait a couple days with a doctor? Personally, I wouldn’t feel comfortable doing that. So that’s where I also use my own clinical reasoning to make those decisions.”* Student 6 * “Management plan? I guess it says that it’s safe to do so. I’ve kind of really stuck to the Framework…used it as the Framework that was intended and regardless, they seem to be fine for PT care.”* Student 8
Personal characteristics	Risk benefit considerations from the Framework supported all participants except one to identify precautions to physiotherapy interventions, which led to an adaptable approach to treatment that prioritized patient safety, and justifying this approach: “*I definitely would not be manipulating at this stage…I would probably stick with mid-range mobilization.”* Student 3 * “The Framework has a part where arterial problems and mobilizations, like there are some more precautions about that, so that’s why don’t do any high velocity movements. But gentle mobilizations as long as pain can be tolerated are, are okay.”* Student 6 Decisions for selecting physiotherapy interventions were shared with the patient for most participants: * “Shared decision making to try to really instill some behavior change, just knowing that she has these all of these comorbidities and fear.”* Student 1 Physiotherapy interventions were individualized to the patient for most participants, and considered the whole person and future risk for vascular pathology: * “Fear of movement is something we’ll definitely have to discuss with the patient.”* Student 7 * “I think we could probably do a little bit better in terms of improving your cardiovascular health”* Student 4 The Framework supported communication with the patient’s family doctor for collaborative patient-centered care for some participants: * “I’m going to write a note to the family doctor anyway saying that we saw her. This has been kind of ruled out. All of the vascular stuff looks good on testing.”* Student 7

## Discussion

This is the first study to explore the influence of the Framework on clinical reasoning processes in postgraduate physiotherapy students. The Framework supported clinical reasoning that used primarily hypothetico-deductive processes. The Framework informed vascular hypothesis generation in the patient history and testing the vascular hypothesis through patient history questions and selection of physical examination tests to inform clarity and support for diagnosis and management. Most postgraduate students’ clinical reasoning processes were characterized by high-level features (e.g. specificity, prioritization). However, some demonstrated occasional difficulties or errors, reflecting a continuum of clinical reasoning proficiency. Clinical reasoning processes were informed by deep knowledge of the Framework integrated with a breadth of wider knowledge and supported by a range of personal characteristics (e.g., justification for decisions, reflection).

### Use of the Framework to inform clinical reasoning processes

The Framework provided a structured and comprehensive approach to support postgraduate students’ clinical reasoning processes in assessment and management of the cervical spine region, considering the potential for vascular pathology. Patient history and physical examination information was evaluated to inform clarity and support the decision to refer for further vascular investigations or proceed with musculoskeletal physiotherapy diagnosis/interventions. The Framework is not intended to lead to a vascular pathology diagnosis [7, 8], and following the Framework does not guarantee vascular pathologies will be identified [41]. Rather, it aims to support a process of clinical reasoning to elicit and interpret appropriate patient history and physical examination information to estimate the probability of vascular pathology and inform judgement about the need to refer for further investigations [7, 8, 42]. Results of this study suggest the Framework has achieved this aim for postgraduate physiotherapy students.

The Framework supported postgraduate students in using primarily hypothetico-deductive diagnostic reasoning processes. This is expected given the diversity of vascular pathology clinical presentations precluding a definite clinical pattern and inherent complexity as a potential masquerader of a musculoskeletal problem [7]. It is also consistent with prior research investigating clinical reasoning processes in musculoskeletal physiotherapy postgraduate students [12] and clinical experts [29] where hypothetico-deductive and pattern recognition diagnostic reasoning are employed according to the demands of the clinical situation [10]. Diagnostic reasoning of most postgraduate students in this study demonstrated features suggestive of high-level clinical reasoning in musculoskeletal physiotherapy [12], including ongoing reasoning with high-level cue identification and interpretation, specificity and prioritization during assessment and treatment, use of physiotherapy interventions to aid diagnostic reasoning, and prognosis determination [12, 29, 43]. Expert physiotherapy practice has been further described as using a dialectical model of clinical reasoning with seamless transitions between clinical reasoning strategies [44]. While diagnostic reasoning was a focus in this study, postgraduate students considered a breadth of information as important to their reasoning (e.g., patient’s perspectives of the reason for their pain). This suggests wider reasoning strategies (e.g., narrative, collaborative) were employed to enable shared decision-making within the context of patient-centered care.

Study findings also highlighted a continuum of proficiency in use of the Framework to inform clinical reasoning processes. Not all students demonstrated all characteristics of high-level clinical reasoning and there are suggestions of incomplete reasoning processes, for example occasional errors in evaluating cues. Some students offered explanations such as incomplete case information as factors contributing to difficulties with clinical reasoning processes. However, the ability to critically evaluate incomplete and potentially conflicting clinical information is consistently identified as an advanced clinical practice competency [14, 43]. A continuum of proficiency in clinical reasoning in musculoskeletal physiotherapy is supported by wider healthcare professions describing acquisition and application of clinical knowledge and skills as a developmental continuum of clinical competence progressing from novice to expert [45, 46]. The range of years of clinical practice experience in this cohort of students (3–14 years) or prior completed postgraduate education may have contributed to the continuum of proficiency, as high-quality and diverse experiential learning is essential for the development of high-level clinical reasoning [14, 47]. 

### Deep knowledge of the Framework informs clinical reasoning processes

Postgraduate students demonstrated deep Framework knowledge to inform clinical reasoning processes. All students demonstrated knowledge of patient history features of vascular pathology, recommended physical examination tests to test a vascular hypothesis, and the need to refer if there is a high level of suspicion for vascular pathology. A key development in the recent Framework update is the removal of the recommendation to perform positional testing [8]. All students demonstrated knowledge of this development, and none wanted to test a vascular hypothesis with positional testing. Most also demonstrated Framework knowledge about considerations for planning treatment with physiotherapy interventions (e.g., risk-benefit analysis, informed consent), though not all, which underscores the continuum of proficiency in postgraduate students. Rich organization of multidimensional knowledge is a required component for high level clinical reasoning and is characteristic of expert physiotherapy practice [10, 48, 49]. Most postgraduate physiotherapy students displayed this expert practice characteristic through integration of deep Framework knowledge with a breadth of prior knowledge (e.g., experiential, propositional) to inform clinical reasoning processes. This highlights the utility of the Framework in postgraduate physiotherapy education to develop advanced level evidence-based knowledge informing clinical reasoning processes for safe assessment and management of the cervical spine, considering the potential for vascular pathology [9, 8, 50–52].

### Framework supports personal characteristics to facilitate integration of knowledge and clinical reasoning

The Framework supported personal characteristics of postgraduate students, which are key drivers for the complex integration of advanced knowledge and high-level clinical reasoning [10, 12, 48]. For all students, the Framework supported justification for decisions and patient-centered care, emphasizing a whole-person approach and shared decision-making. Further demonstrating a continuum of proficiency, the Framework supported a wider breadth of personal characteristics for some students, including critical analysis, reflection, self-analysis, and adaptability. These personal characteristics illustrate the interwoven cognitive and metacognitive skills that influence and support a high level of clinical reasoning [10, 12] and the development of clinical expertise [48, 53]. For example [54], reflection is critical to developing high-level clinical reasoning and advanced level practice [12, 55]. Postgraduate students reflected on prior knowledge, experiences, and action within the context of current Framework knowledge, emphasizing active engagement in cognitive processes to inform clinical reasoning processes. Reflection-in-action is highlighted by self-analysis and adaptability. These characteristics require continuous cognitive processing to consider personal strengths and limitations in the context of the patient and evidence-based practice, adapting the clinical encounter as required [53, 55]. These findings highlight use of the Framework in postgraduate education to support development of personal characteristics that are indicative of an advanced level of clinical practice [12]. 

### Synthesis of findings

**Fig. 2:**
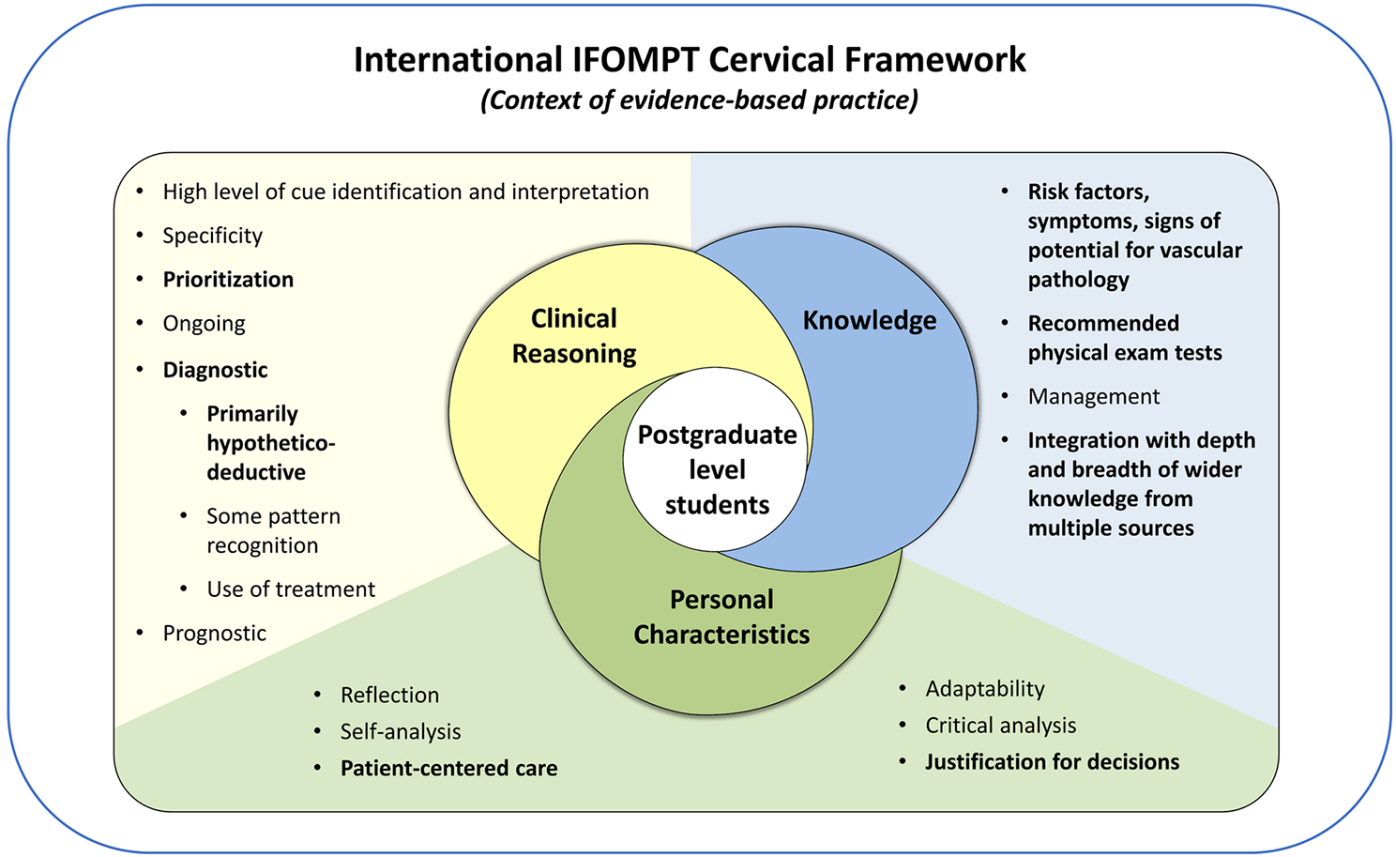
Use of the Framework to inform clinical reasoning in postgraduate level musculoskeletal physiotherapy students. Adapted from the Postgraduate Musculoskeletal Physiotherapy Practice model [12].

Derived from synthesis of research study findings and informed by the Postgraduate Musculoskeletal Physiotherapy Practice model [12], use of the Framework to inform clinical reasoning processes in postgraduate students is illustrated in Fig. 2. Overlapping clinical reasoning, knowledge and personal characteristic components emphasize the complex interaction of factors contributing to clinical reasoning processes. Personal characteristics of postgraduate students underpin clinical reasoning and knowledge, highlighting their role in facilitating the integration of these two components. Bolded subcomponents indicate convergence of results reflecting all postgraduate students and underscores the variability among postgraduate students contributing to a continuum of clinical reasoning proficiency. The relative weighting of the components is approximately equal to balance the breadth and convergence of subcomponents. Synthesis of findings align with the Postgraduate Musculoskeletal Physiotherapy Practice model [12], though some differences exist. Limited personal characteristics were identified in this study with little convergence across students, which may be due to the objective of this study and the case analysis approach.

### Strengths and limitations

Think aloud case analyses enabled situationally dependent understanding of the Framework to inform clinical reasoning processes in postgraduate level students [17], considering the rare potential for vascular pathology. A limitation of this approach was the standardized nature of case information provided to students, which may have influenced clinical reasoning processes. Future research studies may consider patient case simulation to address this limitation [30]. Interviews were conducted during the second half of the postgraduate educational program, and this timing could have influenced clinical reasoning processes compared to if interviews were conducted at the end of the program. Future research can explore use of the Framework to inform clinical reasoning processes in established advanced practice physiotherapists. The sample size of this study aligns with recommendations for think aloud methodology [27, 28], achieved rich data, and purposive sampling enabled wide representation of key characteristics (e.g., gender, ethnicity, country of training, physiotherapy experiences), which enhances transferability of findings. Students were aware of the study objective in advance of interviews which may have contributed to a heightened level of awareness of vascular pathology. The prior relationship between students and researchers may have also influenced results, however several strategies were implemented to minimize this influence.

### Implications

The Framework is widely implemented within IFOMPT postgraduate educational programs and has led to important shifts in educational curricula [9]. Findings of this study support use of the Framework as an educational resource in postgraduate physiotherapy programs to inform clinical reasoning processes for safe and effective assessment and management of cervical spine presentations considering the potential for vascular pathology. Individualized approaches may be required to support each student, owing to a continuum of clinical reasoning proficiency. As the Framework was written for practicing musculoskeletal clinicians, future research is required to explore use of the Framework to inform clinical reasoning in learners at different levels, for example entry-level physiotherapy students.

## Conclusions

The Framework supported clinical reasoning that used primarily hypothetico-deductive processes in postgraduate physiotherapy students. It informed vascular hypothesis generation in the patient history and testing the vascular hypothesis through patient history questions and selection of physical examination tests, to inform clarity and support for diagnosis and management. Most postgraduate students clinical reasoning processes were characterized as high-level, informed by deep Framework knowledge integrated with a breadth of wider knowledge, and supported by a range of personal characteristics to facilitate the integration of advanced knowledge and high-level clinical reasoning. Future research is required to explore use of the Framework to inform clinical reasoning in learners at different levels.

### Electronic supplementary material

Below is the link to the electronic supplementary material.

Supplementary Material 1

Supplementary Material 2

Supplementary Material 3

Supplementary Material 4

## Data Availability

The dataset used and analyzed during the current study are available from the corresponding author on reasonable request.

## Declarations

### Authors’ information

The lead author of this study (AR) is the first author of the International IFOMPT Cervical Framework.
