# Supplementary material for: Use of the International IFOMPT Cervical Framework to inform clinical reasoning in postgraduate level physiotherapy students: a qualitative study using think aloud methodology
Source: BMC Med Educ. 2024 May 2;24:486. doi: 10.1186/s12909-024-05399-x (PMC11064242; doi:10.1186/s12909-024-05399-x)
Supplement: Supplementary file 1 — Supplementary Material 1 [file 12909_2024_5399_MOESM1_ESM.pptx]

## Slide 1
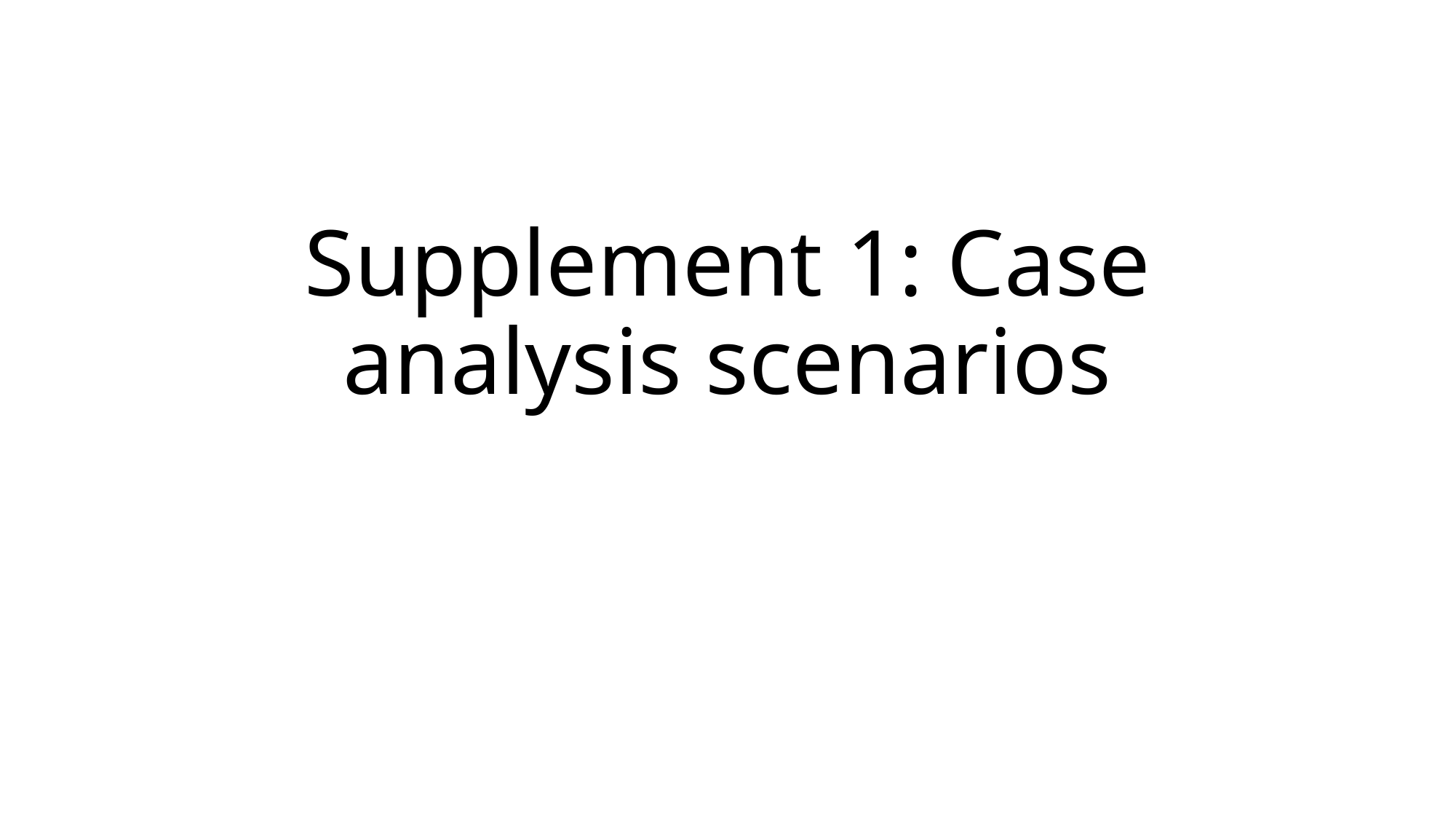

# Supplement 1: Case analysis scenarios

## Slide 2
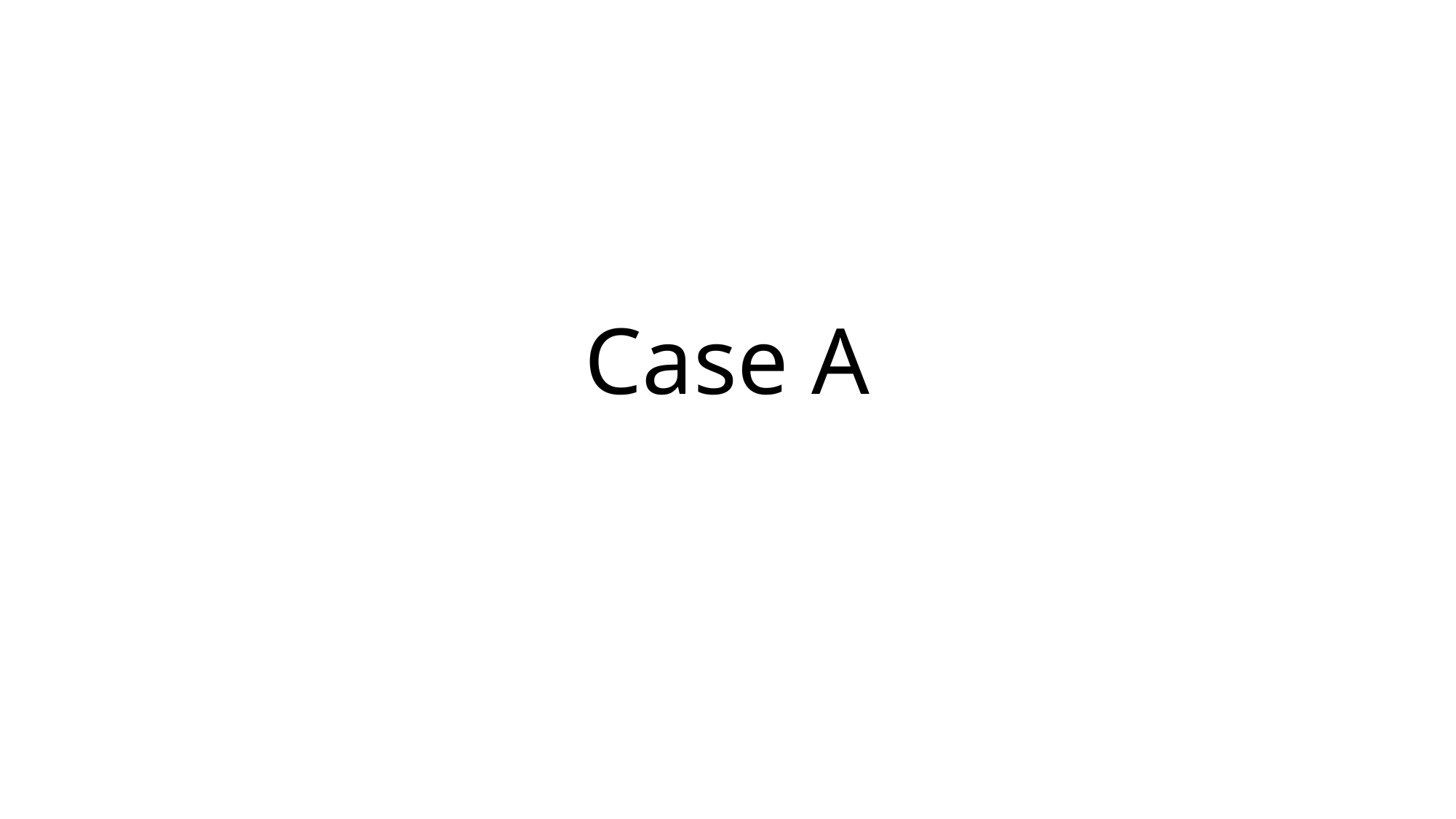

# Case A

## Slide 3
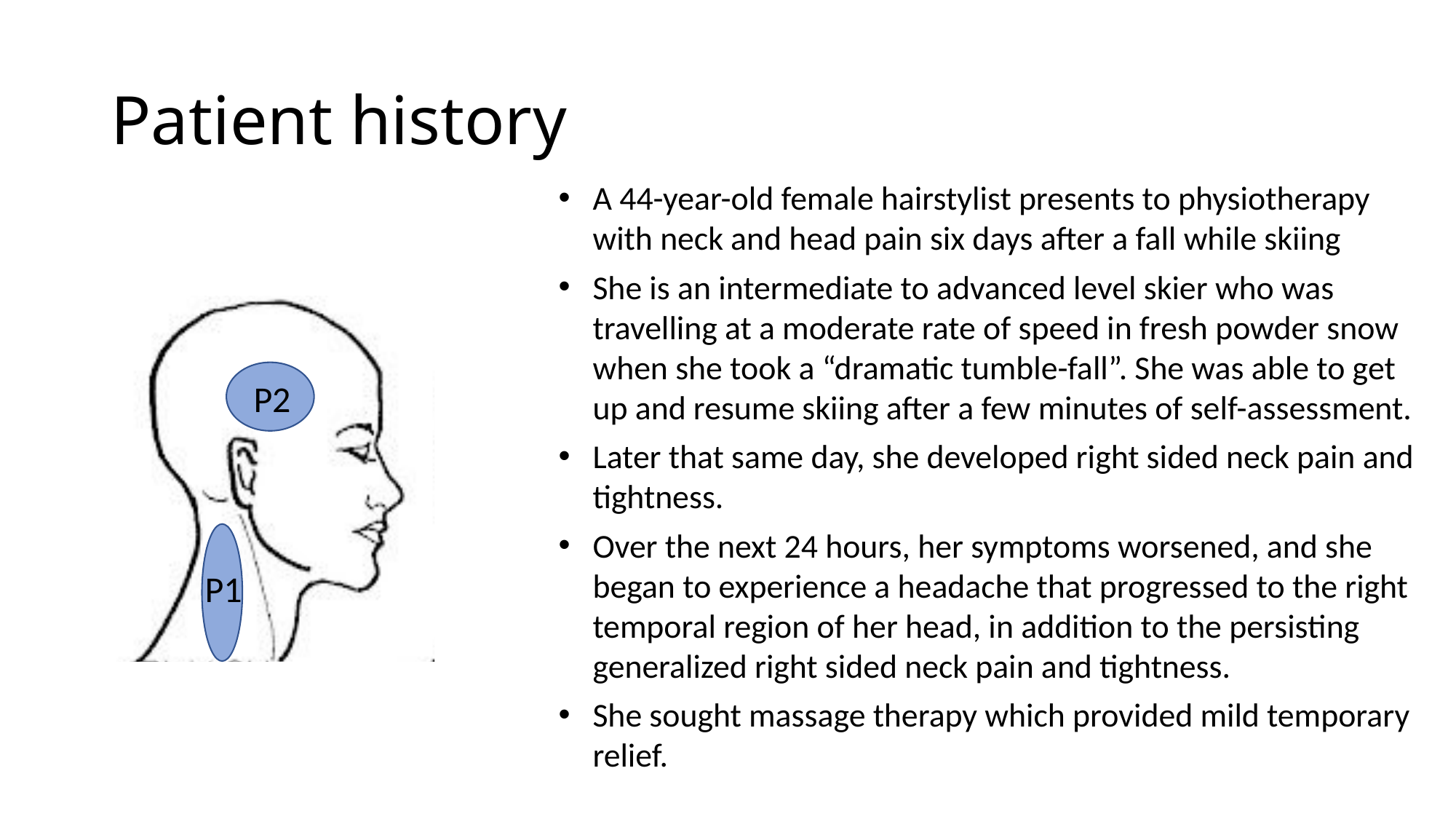

# Patient history
A 44-year-old female hairstylist presents to physiotherapy with neck and head pain six days after a fall while skiing
She is an intermediate to advanced level skier who was travelling at a moderate rate of speed in fresh powder snow when she took a “dramatic tumble-fall”. She was able to get up and resume skiing after a few minutes of self-assessment.
Later that same day, she developed right sided neck pain and tightness.
Over the next 24 hours, her symptoms worsened, and she began to experience a headache that progressed to the right temporal region of her head, in addition to the persisting generalized right sided neck pain and tightness.
She sought massage therapy which provided mild temporary relief.
P2
P1

## Slide 4
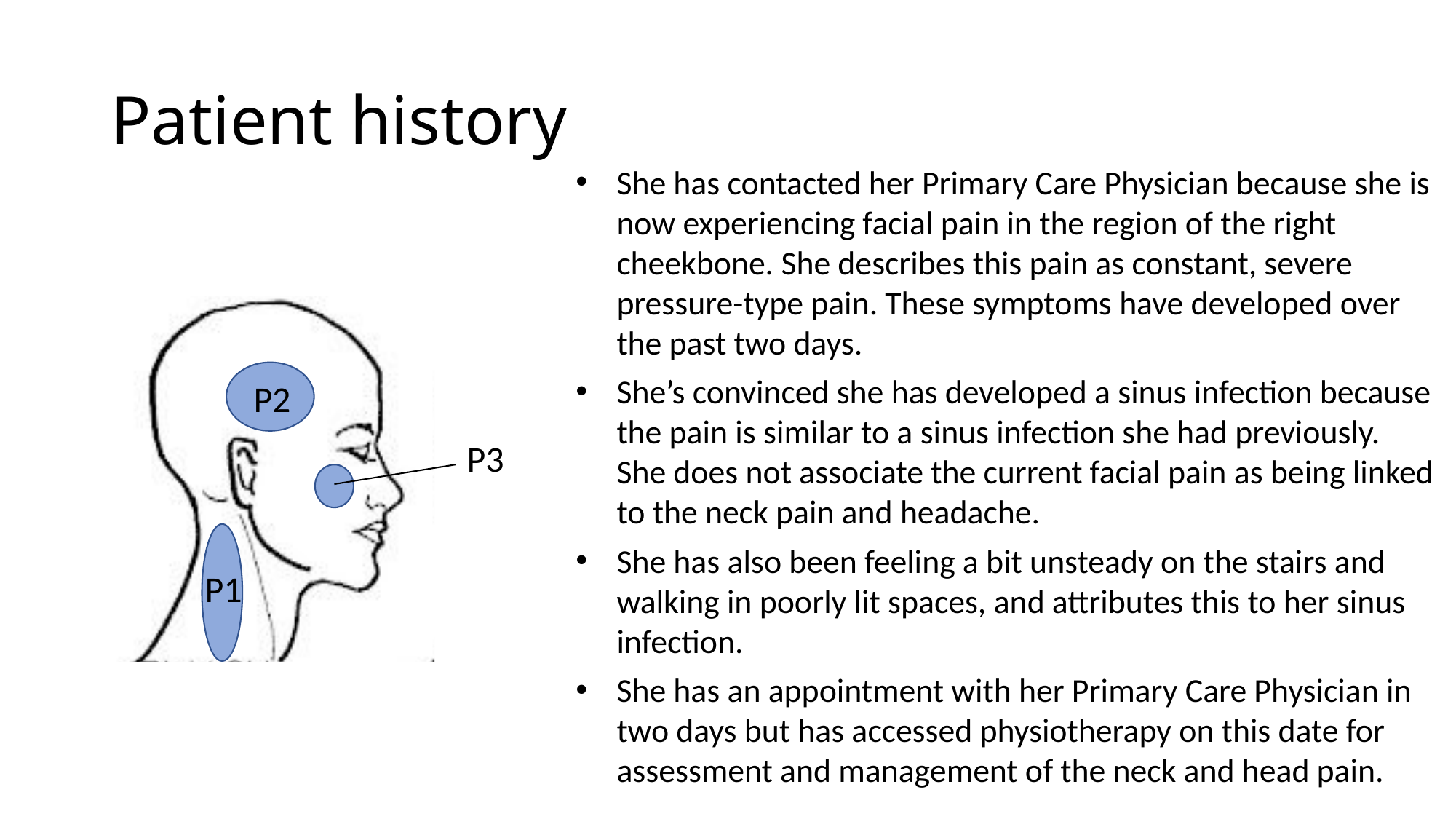

# Patient history
She has contacted her Primary Care Physician because she is now experiencing facial pain in the region of the right cheekbone. She describes this pain as constant, severe pressure-type pain. These symptoms have developed over the past two days.
She’s convinced she has developed a sinus infection because the pain is similar to a sinus infection she had previously. She does not associate the current facial pain as being linked to the neck pain and headache.
She has also been feeling a bit unsteady on the stairs and walking in poorly lit spaces, and attributes this to her sinus infection.
She has an appointment with her Primary Care Physician in two days but has accessed physiotherapy on this date for assessment and management of the neck and head pain.
P2
P3
P1

## Slide 5
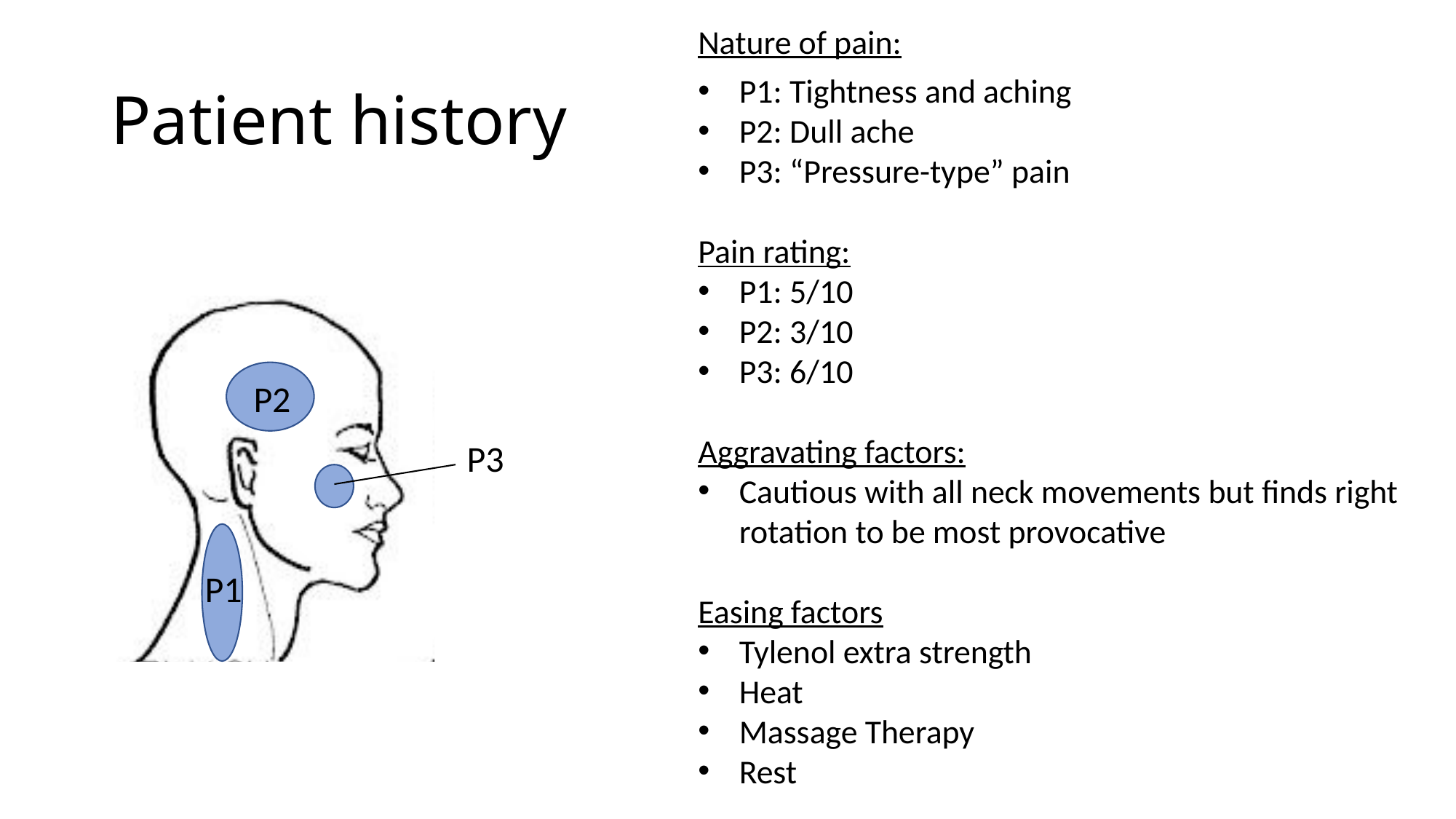

Nature of pain:
P1: Tightness and aching
P2: Dull ache
P3: “Pressure-type” pain
Pain rating:
P1: 5/10
P2: 3/10
P3: 6/10
Aggravating factors:
Cautious with all neck movements but finds right rotation to be most provocative
Easing factors
Tylenol extra strength
Heat
Massage Therapy
Rest
# Patient history
P2
P3
P1

## Slide 6
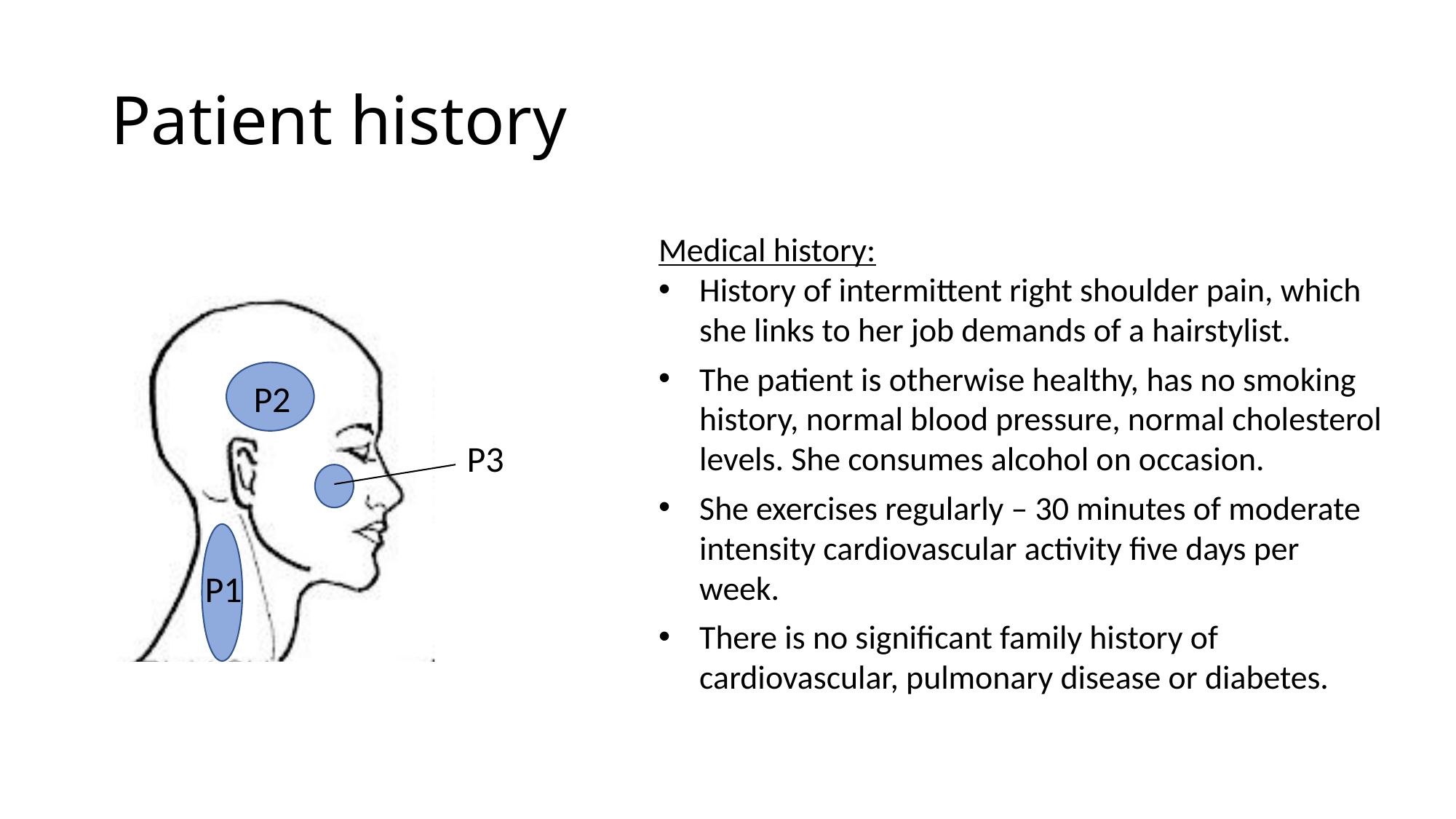

# Patient history
Medical history:
History of intermittent right shoulder pain, which she links to her job demands of a hairstylist.
The patient is otherwise healthy, has no smoking history, normal blood pressure, normal cholesterol levels. She consumes alcohol on occasion.
She exercises regularly – 30 minutes of moderate intensity cardiovascular activity five days per week.
There is no significant family history of cardiovascular, pulmonary disease or diabetes.
P2
P3
P1

## Slide 7
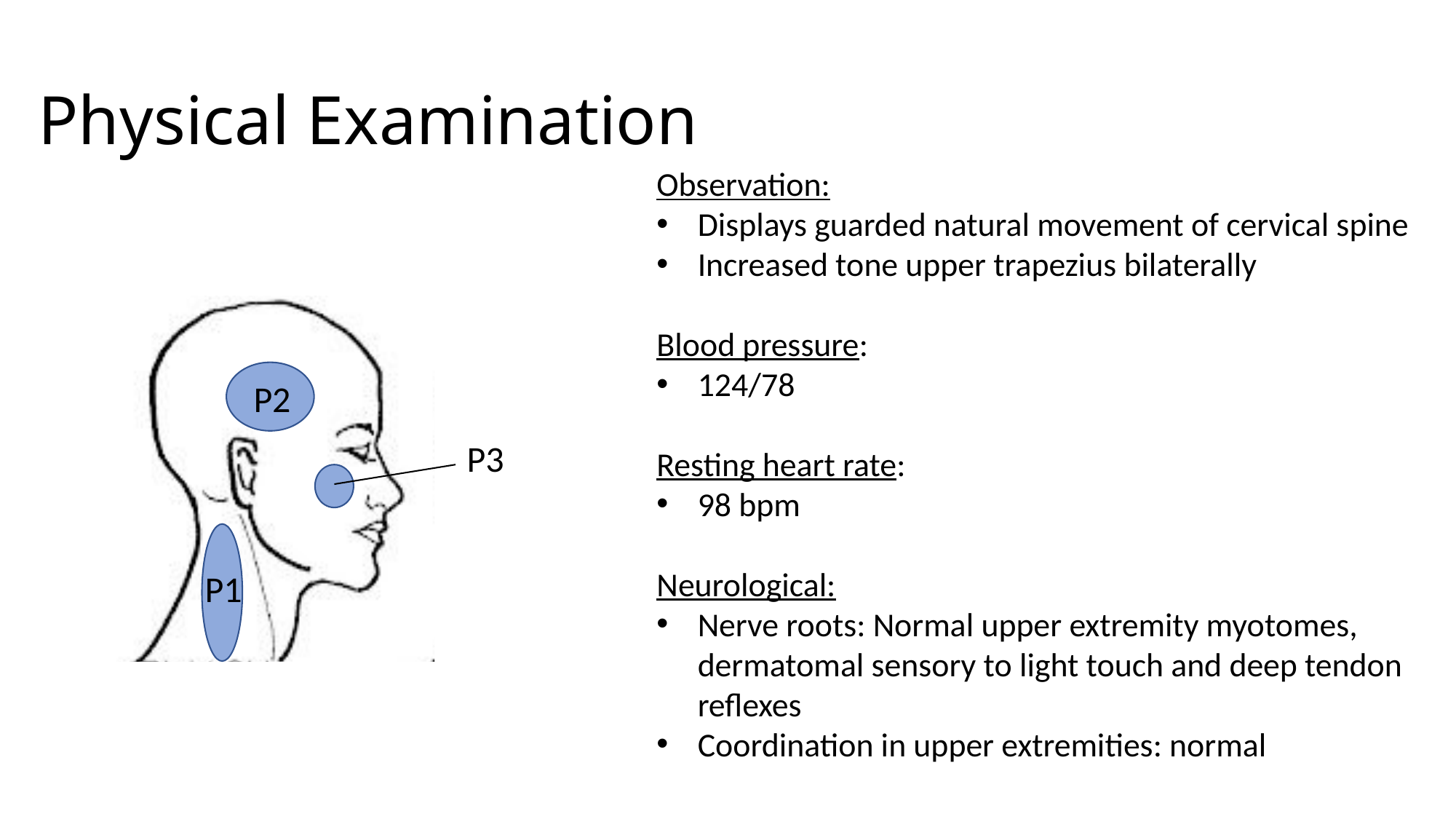

# Physical Examination
Observation:
Displays guarded natural movement of cervical spine
Increased tone upper trapezius bilaterally
Blood pressure:
124/78
Resting heart rate:
98 bpm
Neurological:
Nerve roots: Normal upper extremity myotomes, dermatomal sensory to light touch and deep tendon reflexes
Coordination in upper extremities: normal
P2
P3
P1

## Slide 8
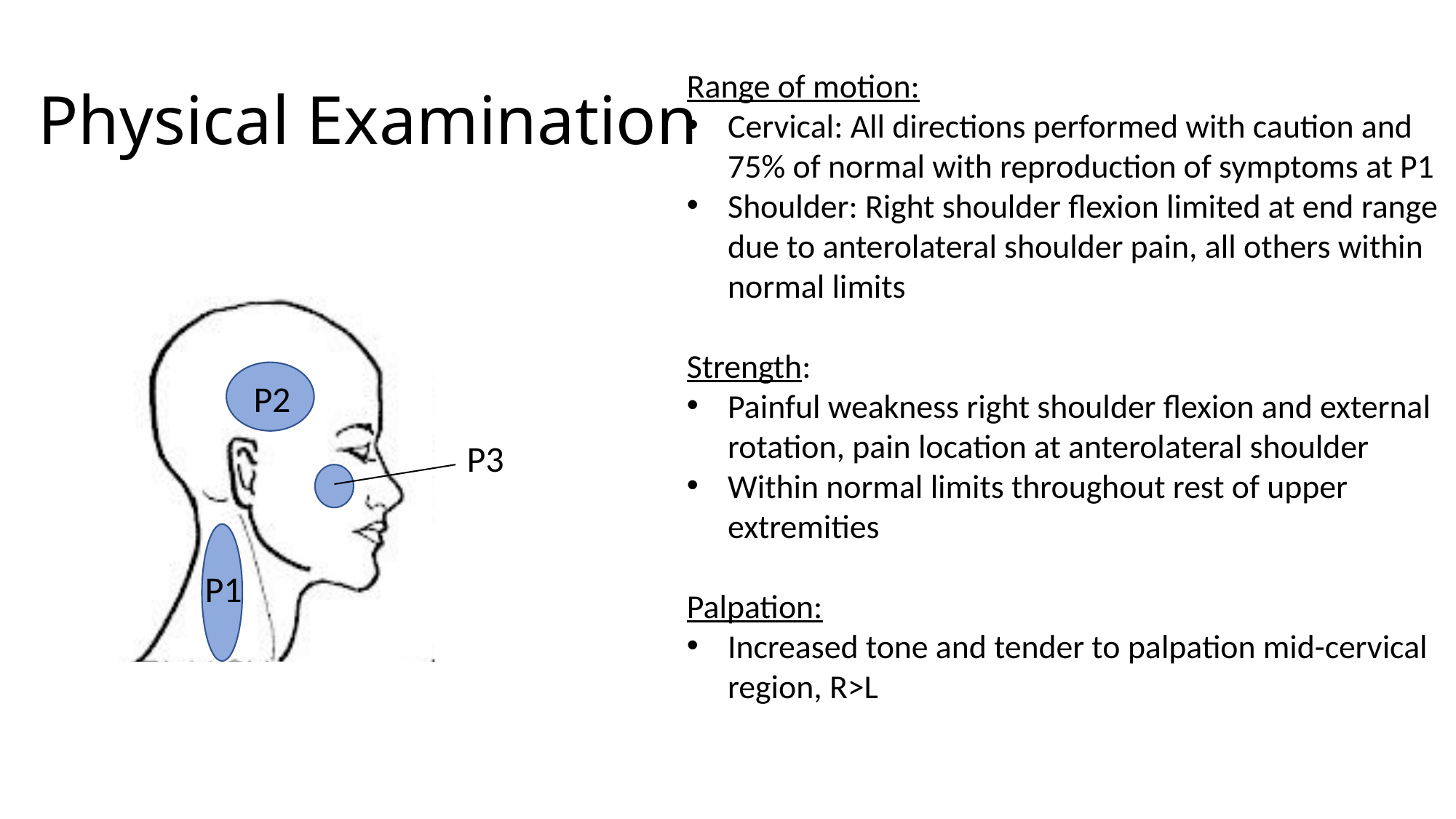

# Physical Examination
Range of motion:
Cervical: All directions performed with caution and 75% of normal with reproduction of symptoms at P1
Shoulder: Right shoulder flexion limited at end range due to anterolateral shoulder pain, all others within normal limits
Strength:
Painful weakness right shoulder flexion and external rotation, pain location at anterolateral shoulder
Within normal limits throughout rest of upper extremities
Palpation:
Increased tone and tender to palpation mid-cervical region, R>L
P2
P3
P1

## Slide 9
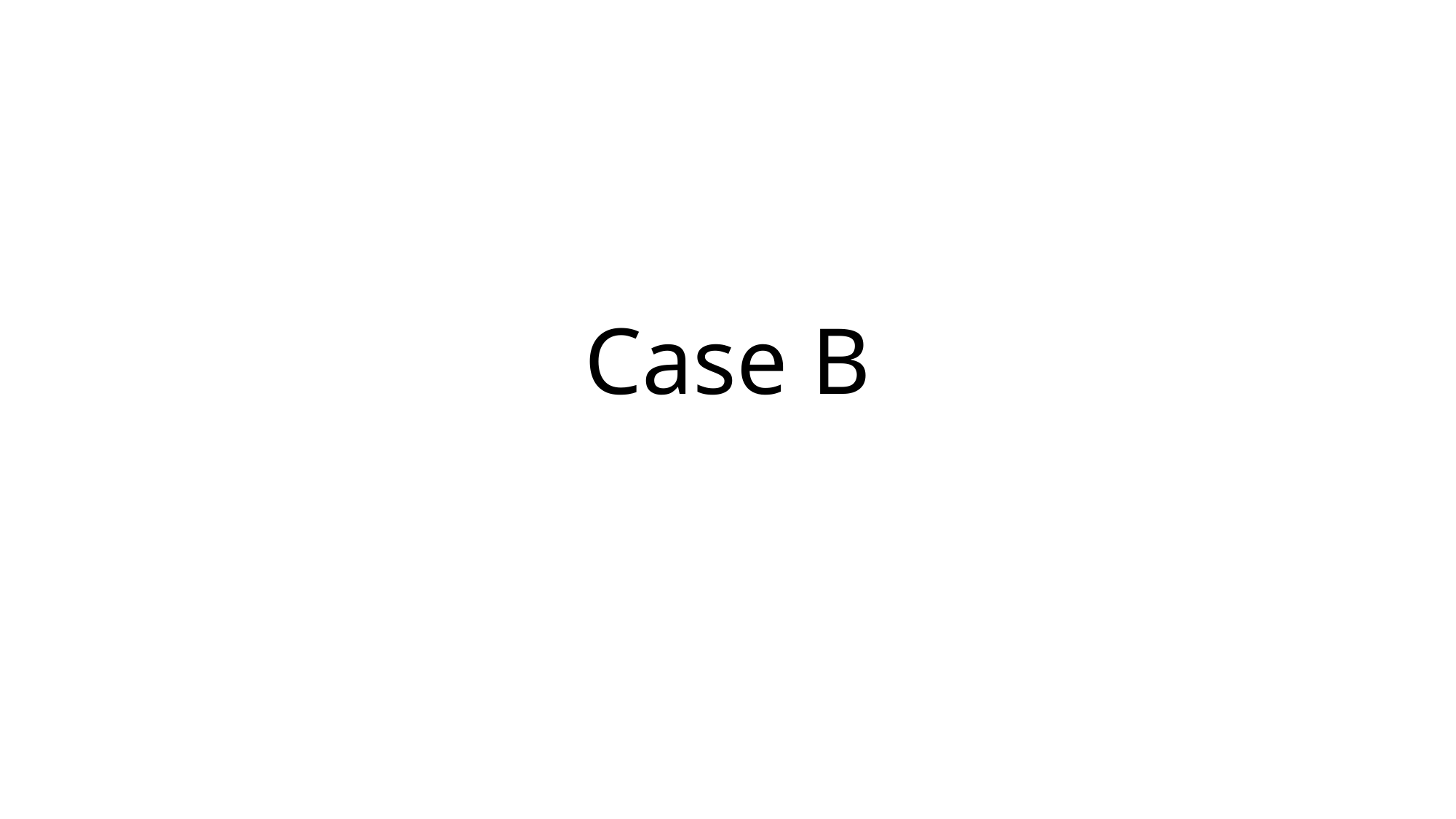

# Case B

## Slide 10
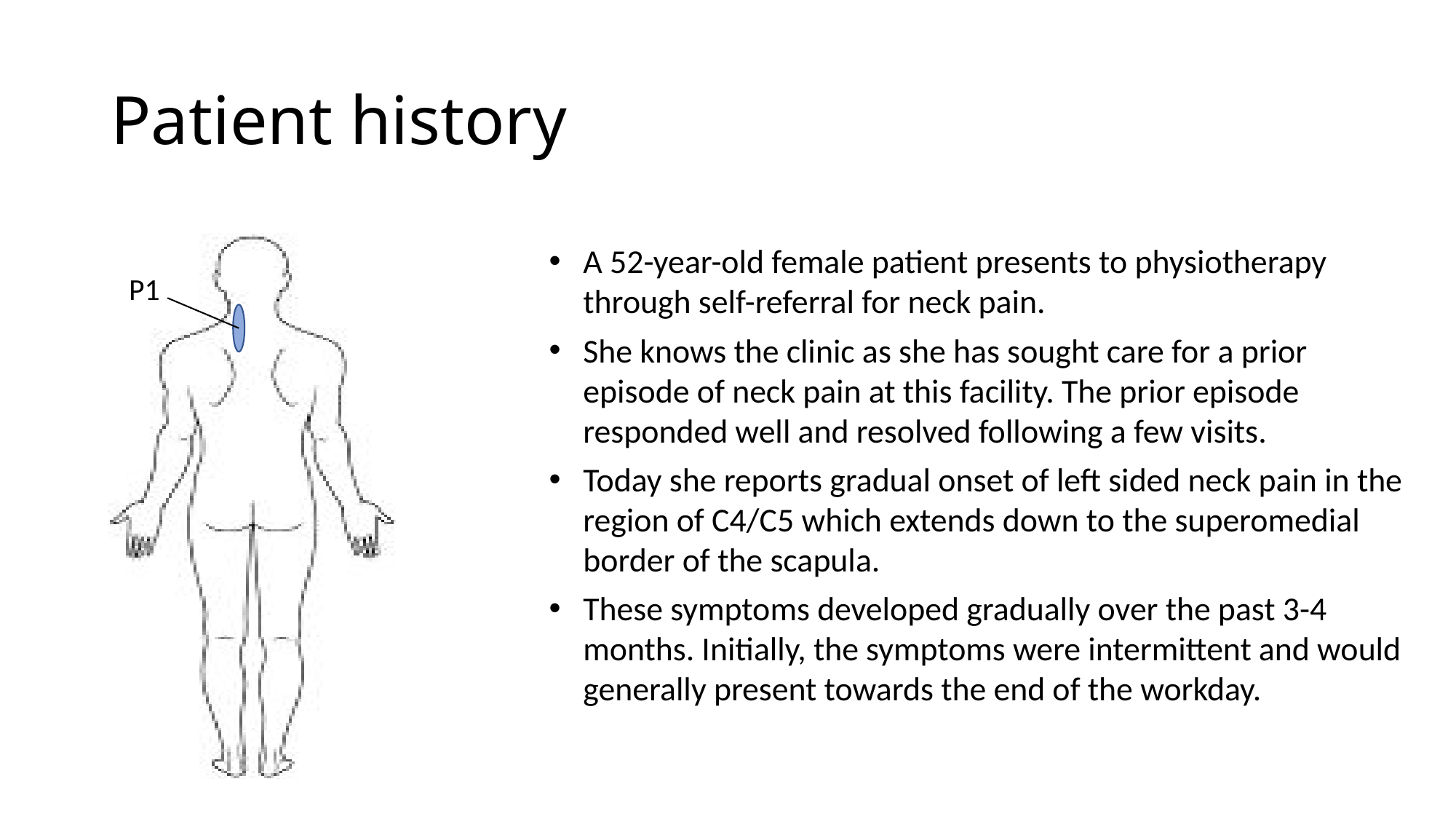

# Patient history
P1
A 52-year-old female patient presents to physiotherapy through self-referral for neck pain.
She knows the clinic as she has sought care for a prior episode of neck pain at this facility. The prior episode responded well and resolved following a few visits.
Today she reports gradual onset of left sided neck pain in the region of C4/C5 which extends down to the superomedial border of the scapula.
These symptoms developed gradually over the past 3-4 months. Initially, the symptoms were intermittent and would generally present towards the end of the workday.

## Slide 11
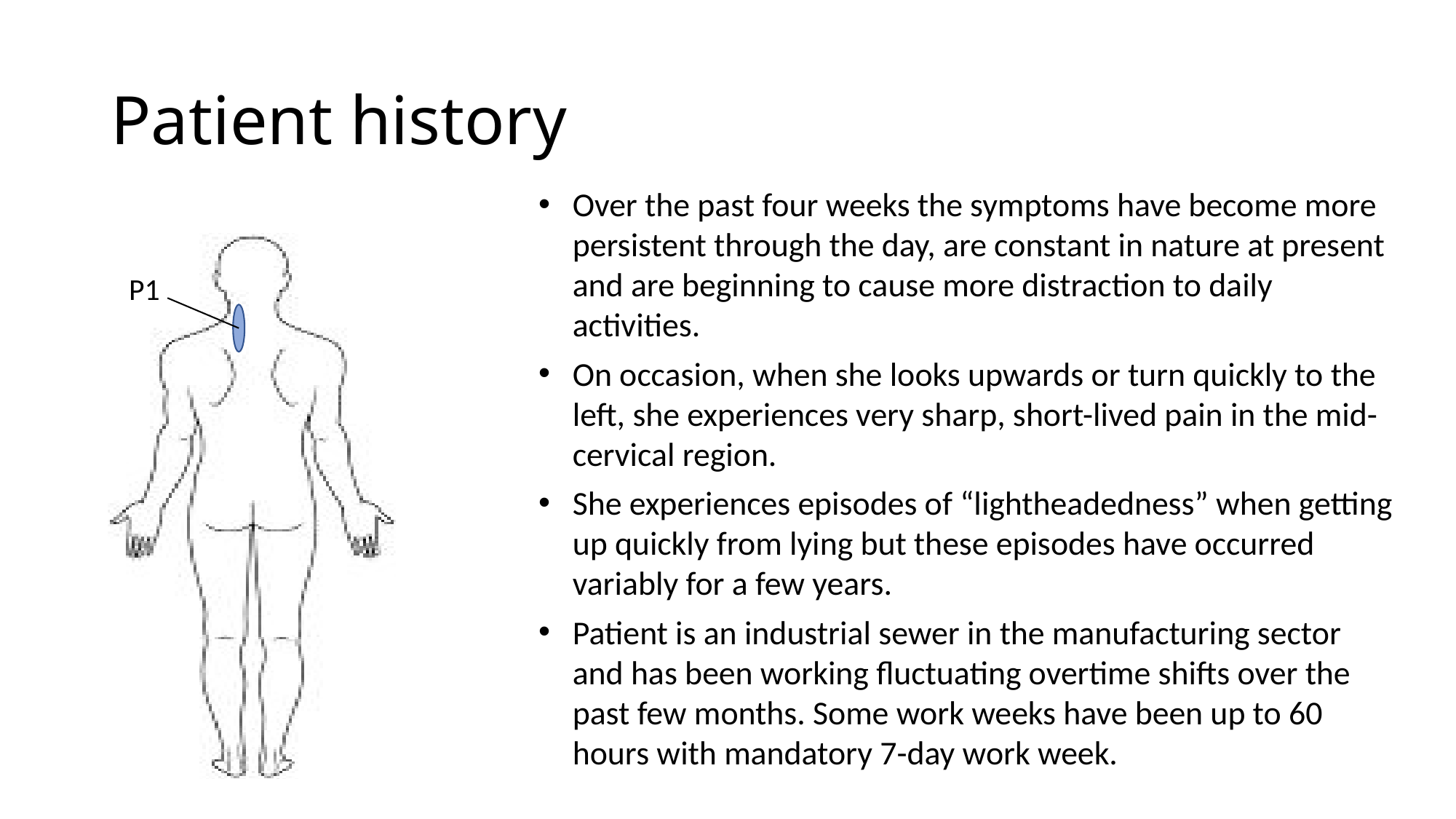

# Patient history
Over the past four weeks the symptoms have become more persistent through the day, are constant in nature at present and are beginning to cause more distraction to daily activities.
On occasion, when she looks upwards or turn quickly to the left, she experiences very sharp, short-lived pain in the mid-cervical region.
She experiences episodes of “lightheadedness” when getting up quickly from lying but these episodes have occurred variably for a few years.
Patient is an industrial sewer in the manufacturing sector and has been working fluctuating overtime shifts over the past few months. Some work weeks have been up to 60 hours with mandatory 7-day work week.
P1

## Slide 12
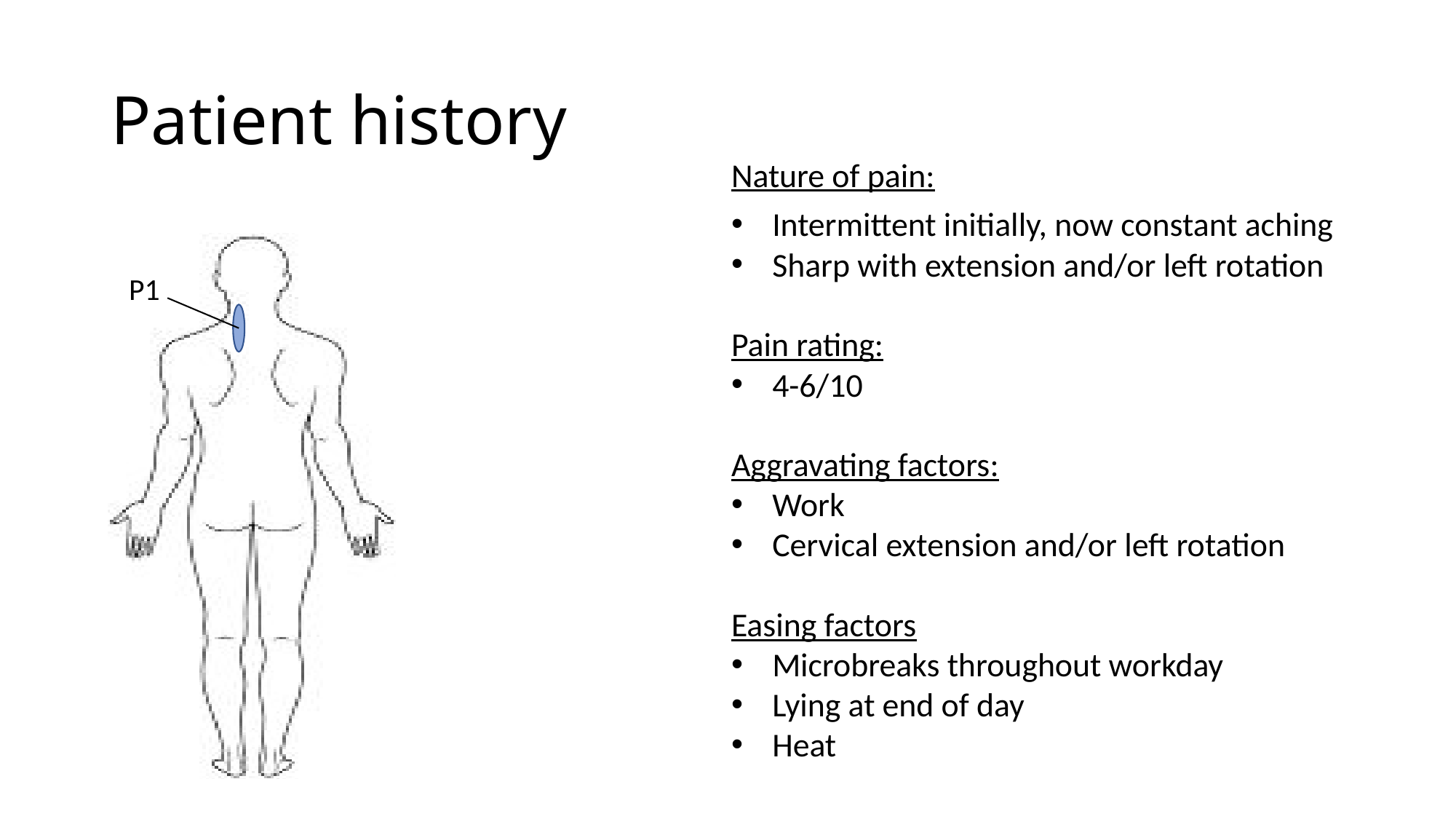

# Patient history
Nature of pain:
Intermittent initially, now constant aching
Sharp with extension and/or left rotation
Pain rating:
4-6/10
Aggravating factors:
Work
Cervical extension and/or left rotation
Easing factors
Microbreaks throughout workday
Lying at end of day
Heat
P1

## Slide 13
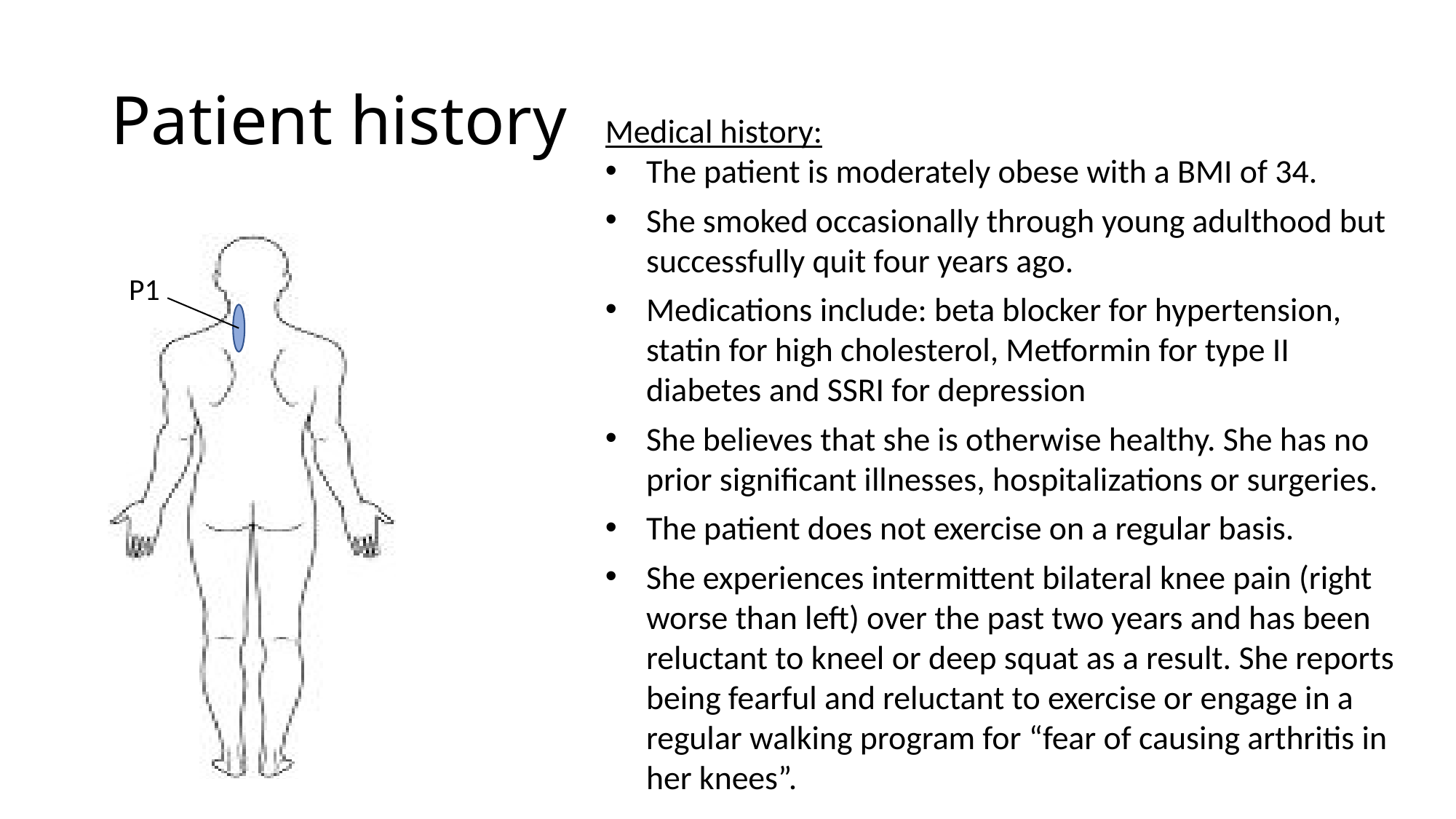

# Patient history
Medical history:
The patient is moderately obese with a BMI of 34.
She smoked occasionally through young adulthood but successfully quit four years ago.
Medications include: beta blocker for hypertension, statin for high cholesterol, Metformin for type II diabetes and SSRI for depression
She believes that she is otherwise healthy. She has no prior significant illnesses, hospitalizations or surgeries.
The patient does not exercise on a regular basis.
She experiences intermittent bilateral knee pain (right worse than left) over the past two years and has been reluctant to kneel or deep squat as a result. She reports being fearful and reluctant to exercise or engage in a regular walking program for “fear of causing arthritis in her knees”.
P1

## Slide 14
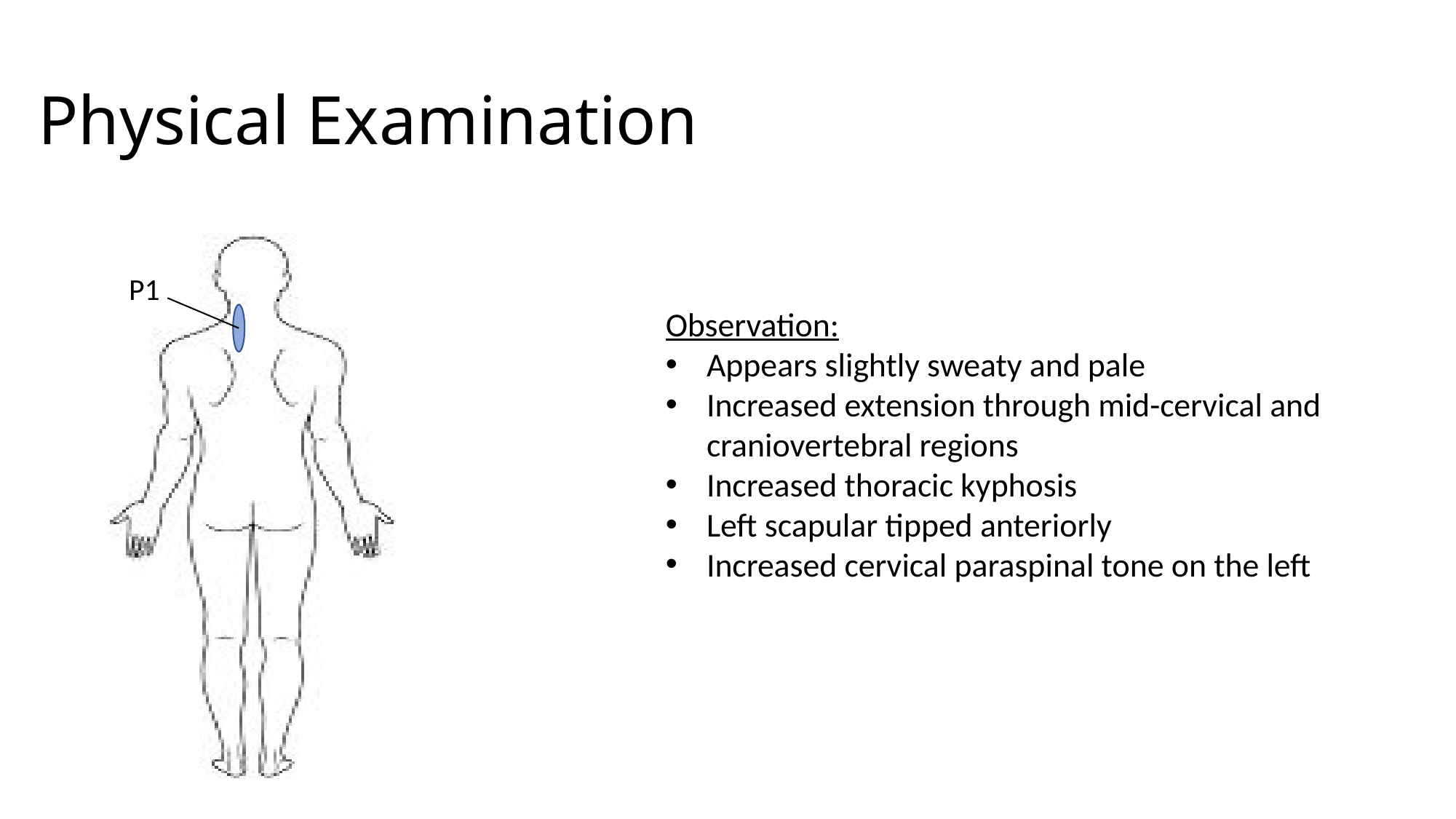

# Physical Examination
P1
Observation:
Appears slightly sweaty and pale
Increased extension through mid-cervical and craniovertebral regions
Increased thoracic kyphosis
Left scapular tipped anteriorly
Increased cervical paraspinal tone on the left

## Slide 15
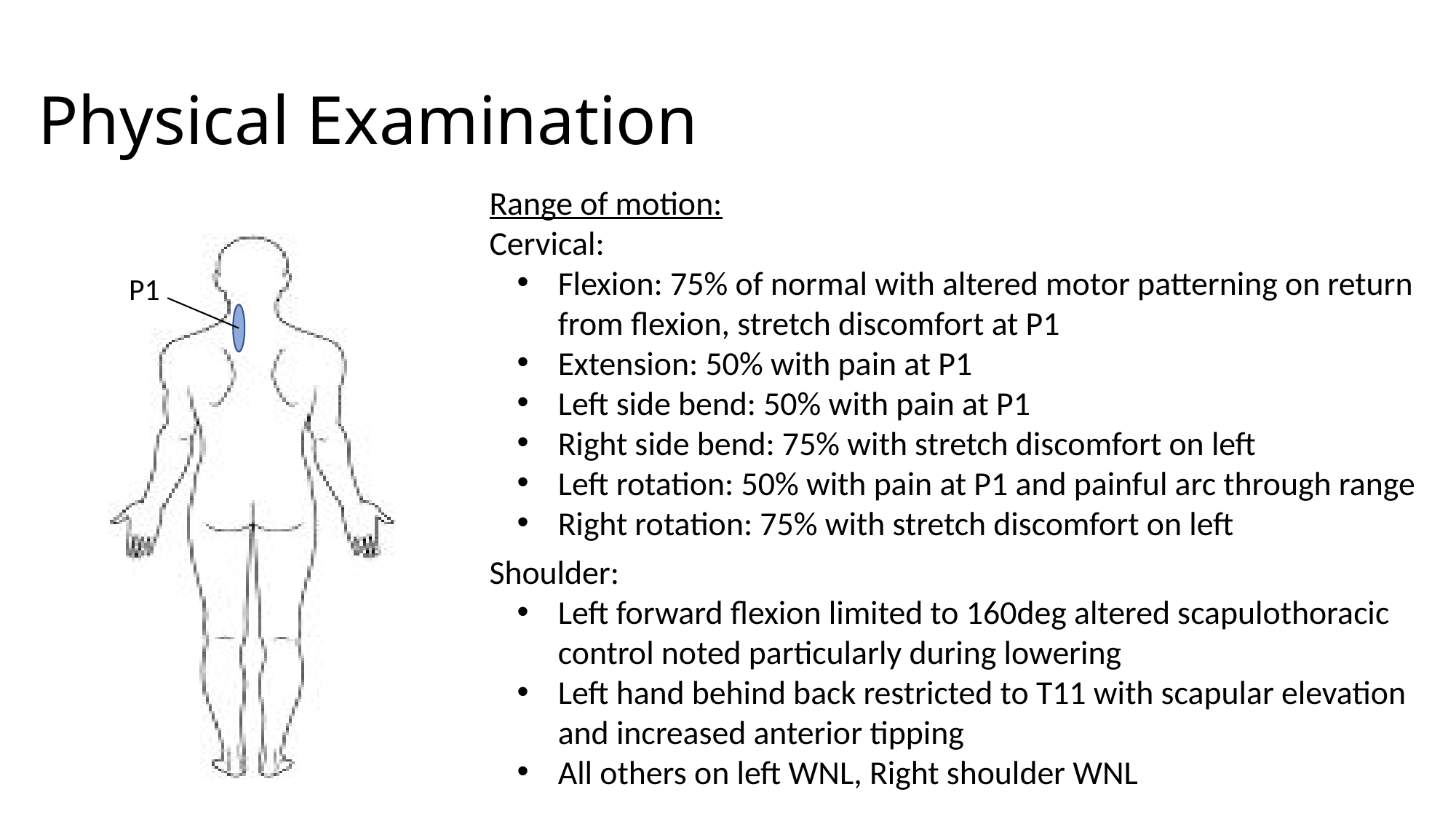

# Physical Examination
Range of motion:
Cervical:
Flexion: 75% of normal with altered motor patterning on return from flexion, stretch discomfort at P1
Extension: 50% with pain at P1
Left side bend: 50% with pain at P1
Right side bend: 75% with stretch discomfort on left
Left rotation: 50% with pain at P1 and painful arc through range
Right rotation: 75% with stretch discomfort on left
Shoulder:
Left forward flexion limited to 160deg altered scapulothoracic control noted particularly during lowering
Left hand behind back restricted to T11 with scapular elevation and increased anterior tipping
All others on left WNL, Right shoulder WNL
P1

## Slide 16
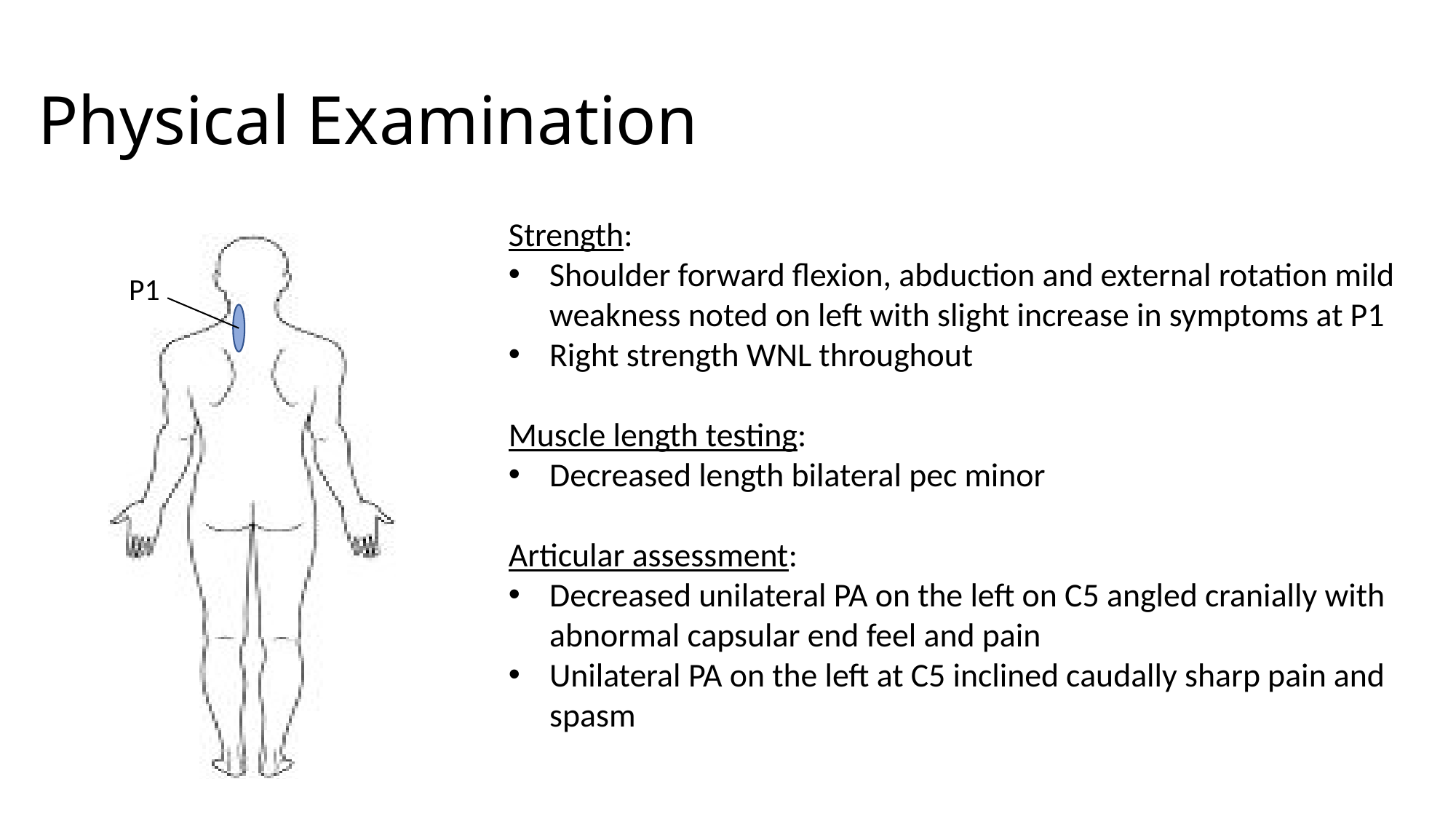

# Physical Examination
Strength:
Shoulder forward flexion, abduction and external rotation mild weakness noted on left with slight increase in symptoms at P1
Right strength WNL throughout
Muscle length testing:
Decreased length bilateral pec minor
Articular assessment:
Decreased unilateral PA on the left on C5 angled cranially with abnormal capsular end feel and pain
Unilateral PA on the left at C5 inclined caudally sharp pain and spasm
P1
