# Supplementary material for: Use of the International IFOMPT Cervical Framework to inform clinical reasoning in postgraduate level physiotherapy students: a qualitative study using think aloud methodology
Source: BMC Med Educ. 2024 May 2;24:486. doi: 10.1186/s12909-024-05399-x (PMC11064242; doi:10.1186/s12909-024-05399-x)
Supplement: Supplementary file 2 — Supplementary Material 2 [file 12909_2024_5399_MOESM2_ESM.docx]

**Supplement 2. Think aloud interview guide**

**Introduction**

[researcher to introduce self]

I would like to thank you for participating in this research study and for joining me today for this think aloud case analysis. As a reminder, this session will be recorded. All information you share will be kept confidential and we anonymize the data. If you wish, you can withdraw from participating in this study at any time. If you withdraw from the interview, you will be asked to indicate if you consent for the use of your data collected up until that point in the interview, or you can direct that all of your data be destroyed. Complete withdrawal of your data from the study will remain an option up until the point of data analysis. If you wish to withdraw at a later date, please contact the researchers, using the contact information in the letter of information. As a reminder, participation in this study will not impact any current or future education or employment opportunities.

We will begin the interview today with a few opening questions so I can have a better understanding of you, your prior physiotherapy experiences and use of the IFOMPT framework for examination of the cervical region for potential vascular pathologies of the neck.

After the opening questions you will work through two separate clinical case scenarios describing your thoughts. When we finish with the first case, we will move onto the second one, each lasting approximately 30-40 min. Case information will be presented verbally and on a PowerPoint slide that I will screen share. For each case, information will be presented to you in stages to reflect the chronology of a patient examination in clinical practice. For example, you will first receive the patient’s clinical history, followed by findings from the physical examination. Throughout the process, you may write down any information you would like to help you work through the cases. I will also be taking notes throughout. As you work through each case, your task is to verbalize out loud your thought processes. You will be asked how you are using the IFOMPT cervical framework to guide your decisions. I will give you further information about this task after the opening questions.

*Questions to ask participant:*

- Before we start do you have any questions?
- Can I confirm that you have read and understand the letter of information and signed the consent form?
- Are you comfortable to proceed?

**Opening questions for think aloud case analysis**

- What is your age?
- What is your gender?
- What is your ethnicity?
- For MPT students:
  - What placements have you completed?
  - What populations of patients do you have experience with?
- For CMP and SEM students:
  - What school did you graduate from and when?
    - Undergraduate or Physiotherapy school as applicable
  - Have you completed post-graduate training? If so, please describe.
  - Please describe your clinical practice experience.
    - Number of years
    - Practice setting
  - Describe, in general, the populations of patients you most commonly assess and treat
- How do you use published frameworks to guide your clinical practice?
- How familiar are you with the IFOMPT framework for examination of the cervical region for potential vascular pathologies of the neck prior to musculoskeletal intervention?
  - When was the last time you reviewed the framework?
  - How do you integrate the framework into your clinical practice when assessing and treating patients presenting with neck and/or head pain?

**Case Introduction**

We will now begin with the clinical case analyses. The cases will follow the structure of the IFOMPT cervical framework including patient history, planning of the physical examination, physical examination, treatment planning and evaluation of management.

Throughout each case analysis, we are interested in your thought processes and how you are using the IFOMPT cervical framework to inform your clinical reasoning. Every time you receive a new piece of case information, we would like to understand what you are thinking and what is influencing those thought processes. To do this, we ask that you say everything out loud as it goes through your mind. Please share any and all thoughts out loud, even if you may not feel they are relevant, including how you are using the IFOMPT cervical framework to inform your clinical reasoning. If you pause for longer than a few seconds, you will be reminded to “keep thinking aloud”. If you have difficulty thinking aloud, we will provide prompting questions to facilitate your thought processes.

For example, as you receive pieces of the patient history, verbalize the clinical hypotheses you are generating, the history features contributing to or refuting those hypotheses and any additional questions you would like to ask. Another example is to verbalize how the patient history is influencing your planning of the physical examination, including presence of contraindications, precautions and order of testing. Throughout each case, we would like to know when and how you are using the IFOMPT cervical framework. As you think aloud, be sure to let me know how you are using the framework to inform your clinical reasoning.

*Question to ask participant:*

- Before we start do you have any questions?

**Case A**

**Patient History Stage 1**

A 44-year-old female hairstylist presents to physiotherapy with neck and head pain six days after a fall while skiing. She is an intermediate to advanced level skier who was travelling at a moderate rate of speed in fresh powder snow when she took a “dramatic tumble-fall”. She was able to get up and resume skiing after a few minutes of self-assessment. Later that same day, she developed right sided neck pain and tightness. Over the next 24 hours, her symptoms worsened, and she began to experience a headache that progressed to the right temporal region of her head, in addition to the persisting generalized right sided neck pain and tightness. She sought massage therapy which provided mild temporary relief.

[pause for think aloud, using prompts below as needed]

She has contacted her Primary Care Physician because she is now experiencing facial pain in the region of the right cheekbone. She describes this pain as constant, severe pressure-type pain. These symptoms have developed over the past two days. She’s convinced she has developed a sinus infection because the pain is similar to a sinus infection she had previously. She does not associate the current facial pain as being linked to the neck pain and headache. She has also been feeling a bit unsteady on the stairs and walking in poorly lit spaces, and attributes this to her sinus infection. She has an appointment with her Primary Care Physician in two days but has accessed physiotherapy on this date for assessment and management of the neck and head pain.

[pause for think aloud, using prompts below as needed]


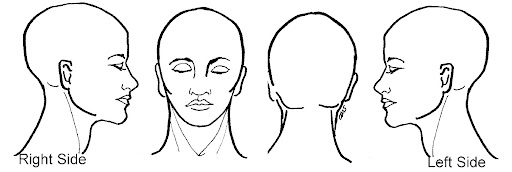


Nature of pain:

P2

P1: Tightness and aching

P2: Dull ache

P3

P3: “pressure-type” pain

P1

Pain rating:

P1: 5/10

P2: 3/10

P3: 6/10

Aggravating factors

- Cautious with all neck movements but finds right rotation to be most provocative

Easing factors

- Tylenol extra strength
- Heat
- Massage Therapy
- Rest

[pause for think aloud, using prompts below as needed]

| **Break for analysis with think aloud – History Stage 1** |
| --- |
| *Question to ask before proceeding:*   - *Is there anything further that you would like to add at this point that you haven’t already said?*   *Prompts if needed:*   - *What are your hypotheses at this stage?* - *What history features are contributing to your hypotheses?* - *How are you using the cervical framework to generate your hypotheses?* - *What additional questions would you like to ask? Why – what is that information telling you?* - *How are you using the cervical framework to guide the patient history?* |

**Patient History Stage 2**

Medical history:

History of intermittent right shoulder pain, which she links to her job demands of a hairstylist. The patient is otherwise healthy, has no smoking history, normal blood pressure, normal cholesterol levels. She consumes alcohol on occasion. There is no significant family history of cardiovascular, pulmonary disease or diabetes. She exercises regularly – 30 minutes of moderate intensity cardiovascular activity five days per week.

| **Break for analysis with think aloud – History Stage 2** |
| --- |
| *Question to ask before proceeding:*   - *Is there anything further that you would like to add at this point that you haven’t already said?*   *Prompts if needed:*   - *Are there any further questions you would like to ask? Why – what is that information telling you?* - *What are your hypotheses at this stage?* - *How did the medical history contribute to or modify your hypotheses?* - *How are you using the cervical framework to generate and rank your hypotheses?* - *Are there any precautions or contraindications to the physical exam?* - *What is your plan for the physical examination, including order of testing?* - *How are you using the cervical framework to inform your planning of the physical examination?* |

**Physical Examination Stage 1**

- Observation:
  - Displays guarded natural movement of cervical spine
  - Increased tone upper trapezius bilaterally
- Blood Pressure:
  - 124/78
- Resting heart rate:
  - 98 bpm
- Neurological:
  - Nerve roots: Normal upper extremity myotomes, dermatomal sensory to light touch and deep tendon reflexes
  - Coordination in upper extremities: normal

| **Break for analysis with think aloud – Exam Stage 1** |
| --- |
| *Question to ask before proceeding:*   - *Is there anything further that you would like to add at this point that you haven’t already said?*   *Prompts if needed:*   - *What are your hypotheses at this stage?* - *How has the physical examination information refined, re-ranked or rejected your hypotheses?* - *How are you using the cervical framework to refine, re-rank or reject your hypotheses in response to physical examination test outcomes?* - *Are there any precautions or contraindications to the rest of the physical examination?* - *Are there any other physical exam tests you would like to carry out? What additional information are you seeking and why?* - *How are you using the cervical framework to adjust your physical examination based on outcomes of tests?* |

**Physical Examination Stage 2**

- ROM:
  - Cervical: All directions performed with caution and 75% of normal with reproduction of symptoms at P1
  - Shoulder: Right shoulder flexion limited at end range due to anterolateral shoulder pain; all others within normal limits

[pause for think aloud, using prompts below as needed]

- Strength:
  - Painful weakness right shoulder flexion and external rotation, pain location at anterolateral shoulder
  - Within normal limits throughout rest of upper extremities

[pause for think aloud, using prompts below as needed]

- Palpation:
  - Increased tone and tender to palpation mid-cervical region, R>L

| **Break for analysis with think aloud – Exam Stage 2 and management** |
| --- |
| *Question to ask before proceeding:*   - *Is there anything further that you would like to add at this point that you haven’t already said?*   *Prompts if needed:*   - *What is your differential diagnosis?* - *How did the cervical framework contribute to the development of your differential diagnosis?* - *Are there any contraindications or precautions to physiotherapy interventions with this patient?* - *What is your management plan? Please provide details.*   - *If referring on for further investigations, what is the urgency of the referral and to whom are you referring?*   - *If keeping for physiotherapy interventions, how and when will you evaluate the effectiveness of your interventions?* - *How is the cervical framework informing your management plan?* - *How will you communicate your examination findings with the patient and your recommendations for management?* - *How is the cervical framework informing your communication with the patient regarding your examination findings and recommendations for management?* - *Is there anything else you would have wanted to ask or test for this patient? Why – what is that information telling you?* |

**End of case**

That concludes Case A.

**Case B**

**Patient History Stage 1**

A 52-year-old female patient presents to physiotherapy through self-referral for neck pain. She knows the clinic as she has sought care for a prior episode of neck pain at this facility. The prior episode responded well and resolved following a few visits. Today she reports gradual onset of left sided neck pain in the region of C4/C5 which extends down to the superomedial border of the scapula. These symptoms developed gradually over the past 3-4 months. Initially, the symptoms were intermittent and would generally present towards the end of the workday.

[pause for think aloud, using prompts below as needed]

Over the past four weeks the symptoms have become more persistent through the day, are constant in nature at present and are beginning to cause more distraction to daily activities. On occasion, when she looks upwards or turn quickly to the left, she experiences very sharp, short-lived pain in the mid-cervical region. She experiences episodes of “lightheadedness” when getting up quickly from lying but these episodes have occurred variably for a few years.


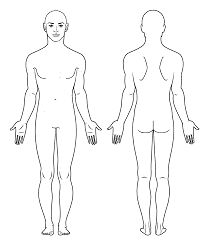
The patient is an industrial sewer in the manufacturing sector and has been working fluctuating over-time shifts over the past few months. Some work weeks have been up to 60 hours with mandatory 7-day work week.

P1

[pause for think aloud, using prompts below as needed]

Nature of pain:

- Intermittent initially, now constant aching
- Sharp with extension and/or left rotation

Pain rating:

- 4-6/10

Aggravating factors

- Work
- Cervical extension and/or left rotation

Easing factors

- Microbreaks throughout workday
- Lying at end of day
- Heat

| **Break for analysis with think aloud – History Stage 1** |
| --- |
| *Question to ask before proceeding:*   - *Is there anything further that you would like to add at this point that you haven’t already said?*   *Prompts if needed:*   - *What are your hypotheses at this stage?* - *What history features are contributing to your hypotheses?* - *How are you using the cervical framework to generate your hypotheses?* - *What additional questions would you like to ask? Why – what is that information telling you?* - *How are you using the cervical framework to guide the patient history?* |

**Patient History Stage 2**

Medical history:

The patient is moderately obese with a BMI of 34. She smoked occasionally through young adulthood but successfully quit four years ago. Medications include: beta blocker for hypertension, statin for high cholesterol, Metformin for type II diabetes and SSRI for depression. She believes that she is otherwise healthy. She has no prior significant illnesses, hospitalizations, or surgeries. The patient does not exercise on a regular basis. She experiences intermittent bilateral knee pain (right worse than left) over the past two years and has been reluctant to kneel or deep squat as a result. She reports being fearful and reluctant to exercise or engage in a regular walking program for “fear of causing arthritis in her knees”.

| **Break for analysis with think aloud – History Stage 2** |
| --- |
| *Question to ask before proceeding:*   - *Is there anything further that you would like to add at this point that you haven’t already said?*   *Prompts if needed:*   - *Are there any further questions you would like to ask? Why – what is that information telling you?* - *What are your hypotheses at this stage?* - *How did the medical history contribute to or modify your hypotheses?* - *How are you using the cervical framework to generate and rank your hypotheses?* - *Are there any precautions or contraindications to the physical exam?* - *What is your plan for the physical examination, including order of testing?* - *How are you using the cervical framework to inform your planning of the physical examination?* |

**Physical Examination**

- Observation:
  - Appears slightly sweaty and pale
  - Increased extension through mid-cervical and craniovertebral regions
  - Increased thoracic kyphosis
  - Left scapula tipped anteriorly
  - Increased cervical paraspinal tone on the left

[pause for think aloud, using prompts below as needed]

- ROM:
  - Cervical:
    - Flexion: 75% of normal with altered motor patterning on return from flexion, stretch discomfort at P1
    - Extension: 50% with pain at P1
    - Left side bend: 50% with pain at P1
    - Right side bend: 75% of normal with stretch discomfort at P1
    - Left rotation: 50% with pain at P1 and painful arc through range
    - Right rotation: 75% of normal with stretch discomfort at P1
  - Shoulder:
    - Left forward flexion limited to 160deg with altered scapulothoracic control noted particularly during lowering
    - Left hand behind back restricted to T11 with scapular elevation and increased anterior tipping
    - All others on left WNL, Right shoulder WNL

[pause for think aloud, using prompts below as needed]

- Strength
  - Shoulder forward flexion, abduction and external rotation mild weakness noted on left with slight increase in symptoms at P1
  - Right strength WNL throughout
- Muscle length testing:
  - Decreased length bilateral pec minor

[pause for think aloud, using prompts below as needed]

- Articular assessment:
  - Decreased unilateral PA on the left on C5 angled cranially with abnormal capsular end feel and pain
  - Unilateral PA on the left at C5 inclined caudally sharp pain and spasm

| **Break for analysis with think aloud – Exam and management** |
| --- |
| *Question to ask before proceeding:*   - *Is there anything further that you would like to add at this point that you haven’t already said?*   *Prompts if needed:*   - *What is your differential diagnosis?* - *How did the cervical framework contribute to the development of your differential diagnosis?*   - *How did you use the cervical framework to refine, re-rank or reject your hypotheses in response to physical examination test outcomes?* - *Are there any contraindications or precautions to physiotherapy interventions with this patient?* - *What is your management plan? Please provide details.*   - *If referring on for further investigations, what is the urgency of the referral and to whom are you referring?*   - *If keeping for physiotherapy interventions, how and when will you evaluate the effectiveness of your interventions?* - *How is the cervical framework informing your management plan?* - *How will you communicate your examination findings with the patient and your recommendations for management?* - *How is the cervical framework informing your communication with the patient regarding your examination findings and recommendations for management?* - *Is there anything else you would have wanted to ask or test for this patient? Why – what is that information telling you?* |

**End of case**

That concludes the analysis of case B.

**End of two cases**

Thank you for your time today. We appreciate it. The next step in the study is to participate in a semi-structured interview. In this interview we will ask you broad questions to explore your perceptions of the value of the IFOMPT cervical framework to inform your clinical reasoning. We would like to schedule this meeting in 2-4 weeks from today.

When are you free to schedule this second interview? [researcher and participant to schedule second interview].

Do you have any final questions for me?
