# Supplementary material for: Use of the International IFOMPT Cervical Framework to inform clinical reasoning in postgraduate level physiotherapy students: a qualitative study using think aloud methodology
Source: BMC Med Educ. 2024 May 2;24:486. doi: 10.1186/s12909-024-05399-x (PMC11064242; doi:10.1186/s12909-024-05399-x)
Supplement: Supplementary file 3 — Supplementary Material 3 [file 12909_2024_5399_MOESM3_ESM.docx]

Supplement 3. Illustrative example of diagnostic reasoning processes

| **Student 6** | | |
| --- | --- | --- |
|  | **Case A** | **Case B** |
| Cues would like to acquire | *None (3 hypotheses generated immediately and all patient history question were used to test these hypotheses).* | Comparison of current pain to previous experience (e.g., location, intensity, onset)? Details about nature of pain on work compared to non-work days? Cardiac history? Any other interventions received? |
| Patient history cues identified | Traumatic mechanism of injury, pain features (location, severity, descriptors), aggravating and alleviating factors, progressing symptoms, unsteadiness, healthy medical history | Working hours, job demands, absence of symptoms into upper extremity, duration and timing of lightheadedness symptoms, gradual onset that is progressing, pain features (descriptor of sharp, intensity), aggravating and alleviating factors, medical history (hypertension, diabetes mellitus, obesity, depression, cholesterol, smoking) |
| Hypotheses generated | Presenting symptoms: fracture, muscle spasm, arterial dissection  Medical history: *none*  Physical exam: *none* | Presenting symptoms: cervical joints, sudden drop in blood pressure for lightheadedness  Medical history: vascular problem  Physical exam: stress fracture, hypoglycemic for observation of sweaty and pale |
| Patient history questions would like to ask to test hypotheses | Does neck movement lead to lightheadedness or imbalance? Past medical history (smoking, stress, medications, hypertension)? Return to exercise? If so, what is response – any change in symptoms during or after? | Presence of numbness or tingling for segmental neurological involvement? Presence of headache, and if so location and pattern of onset? Details of lifestyle and medical history (cardiac history, imaging, medications)? Imaging completed? Would like to use patient reported outcome measures to identify potential yellow flags. |
| Planned physical examination tests would like to perform to test hypotheses | Observation (coloring, inspection of face, asymmetry in function), cervical spine palpation, pulses (radial, carotid), blood pressure, cranial nerves and then if normal continue to range of motion and strength. Still wanting to evaluate cranial nerves and balance at end of case. | Observation (posture, positioning, presence of deformity), palpation (muscle tone, pain response), range of motion of neck and shoulder, pain response to range of motion, muscle function in neck and upper extremities, especially C4-5 myotomal weakness. |
| Suspected diagnosis | Arterial problem | Osteoarthritis related cervical facet joint pain |
| Treatment | Educate on exam findings and refer to emergency room. | Keep for physiotherapy interventions: Educate about condition including normalizing osteoarthritis, movement as beneficial, coping with pain and sleep hygiene, engage to be more active, consider needling for muscle tone but inquire to see how patient feels about this, gentle mobilizations out of end range, no manipulation because of medical history risk factors, inquire if have access to massage treatments.  Response to treatment serves as hypothesis evaluation. If response is improvement, progress to strengthening of shoulder and neck and improving thoracic extension. If response is worsening symptoms, refer for further arterial investigations. |
