# Supplementary material for: Use of the International IFOMPT Cervical Framework to inform clinical reasoning in postgraduate level physiotherapy students: a qualitative study using think aloud methodology
Source: BMC Med Educ. 2024 May 2;24:486. doi: 10.1186/s12909-024-05399-x (PMC11064242; doi:10.1186/s12909-024-05399-x)
Supplement: Supplementary file 4 — Supplementary Material 4 [file 12909_2024_5399_MOESM4_ESM.docx]

Supplement 4. Hypothesis evaluation at each stage of case analyses

|  | **Case A** | **Case B** |
| --- | --- | --- |
| Patient history: Presenting symptoms | A high level of suspicion for vascular pathology was evaluated by all participants:  *“It becomes a bit more concerning right now. First of all, like severe pain after a head injury that comes later, that doesn't, it's not a good sign. So the arterial problem…becomes more important right now. That's that, at this point is the primary hypothesis.”* Student 6 | A high level of suspicion for cervical articular hypotheses was evaluated by all participants. The clinical presentation was familiar enough to four participants, who used pattern recognition:  *“This is already starting to look a lot more like a typical kind of presentation on mechanical neck pain that's...it seems to be pretty localize to the C four, five region moving down to the medial border that scapula, which is still referral pattern for C four, five facet developed over the past three or four months…looks or sounds like it's more of our convergent patterns.”* Student 1  A low-level suspicion for vascular pathology was evaluated by four participants:  *“The third point also further supports the frequent change in position for more vascular event…If there is a vascular issue that is a non-dissecting type.”* Student 5 |
| Patient history: Medical history | The healthy medical history was evaluated against the vascular hypothesis by all participants. Half retained a high level of suspicion for vascular and half lowered their level of suspicion, yet maintained some concern:  *“If it was a no mechanism of injury, then certainly it would decrease my suspicion. Because it was traumatic, so I can't let it go yet.”* Student 4 | The medical history co-morbidities and medication use was evaluated by all participants. Vascular hypotheses remained remote, given the high level of suspicion for cervical articular hypotheses:  *“The medical history made me think about her risk factors...I think it just contributes to my treatment…I don't think she has like a vascular insult right now.”* Student 3 |
| Physical examination | The physical examination shifted the level of suspicion for vascular pathology to a low level in six participants, leading to musculoskeletal diagnoses (i.e., whiplash, cervicogenic pain, facet joint irritation and myofascial contributions):  *“I'm still with the kind of whiplash injury, more mechanical with, like P2 being referred pain because it is unilateral. Doesn't seem to be worsening, P3, maybe facial pain, like say coming from some trigeminal nuclei knowing that traps are all flared up.”* Student 1  A high level of suspicion for vascular pathology was retained through the physical examination by two participants:  *“Based on the Canadian C spine rules because 75% is more than 45 degrees. So we'll go we'll continue with that fracture one is kind of lower priority now…I will continue with my hypothesis for arterial problem.”* Student 6 | The physical examination supported cervical articular hypotheses for all participants:  *“Showing a very clear, articular presentation.”* Student 5  One participant also had a high level of concern for the patient’s appearance within the context of the medical history, which elevated the level of suspicion for vascular pathology:  *“Her being pale and sweaty is just increasing me towards that vascular even further, even knowing that like her objective or her physical examinations still, I mean, she still is presenting with, you know, a degenerative convergent pattern, which she very well probably still has, it's just the vascular one is the more concerning one that we want to make sure we don't miss.”* Student 2 |
